# Mechanism-Matched Pharmacology of Resistance in EGFR-Mutant NSCLC: Genomic Bypass, Lineage Plasticity, Antibody-Drug Conjugates, and Candidate Biophysical Targets

**DOI:** 10.3390/ijms27219569

**Published:** 2026-10-26

**Authors:** Yohan Seo

**Affiliations:** Department of Physiology, Dongguk University College of Medicine, 123 Dongdae-ro, Gyeongju-si 38066, Gyeongsangbuk-do, Republic of Korea; ddukdae12@dongguk.ac.kr; Tel.: +82-54-770-2416

**Keywords:** EGFR, osimertinib, NSCLC, acquired resistance, MET, antibody-drug conjugate, lineage plasticity, drug-tolerant persisters, ion channels

## Abstract

Epidermal growth factor receptor (EGFR)-mutant non-small cell lung cancer (NSCLC) often responds to EGFR tyrosine kinase inhibitors but develops heterogeneous resistance. This structured narrative review integrates clinical and experimental evidence on resistance after osimertinib and intensified EGFR-directed therapy. It distinguishes on-target EGFR alterations, MET and ERBB-family bypass, downstream signaling, oncogenic fusions, histologic transformation, epithelial–mesenchymal plasticity, and drug-tolerant persistence, linking each mechanism to an appropriate assay and treatment context. We evaluate next-generation EGFR inhibitors, EGFR-MET combinations, bispecific antibodies, platinum-pemetrexed intensification, and antibody-drug conjugates directed at HER3, TROP2, or HER2. Clinical validation is assessed separately from mechanistic causality, with attention to allelic phase, central nervous system exposure, target engagement, unbound drug concentration, predictive biomarkers, and safety. Ion channels and transporters, including ANO1/TMEM16A, LRRC8/VRAC, KCa3.1, hERG, STIM-ORAI, TRP, and pH regulators, remain exploratory candidate targets. Available evidence for their role in acquired osimertinib resistance is largely indirect; expression or general tumor biology does not establish disease-specific dependency. A mechanism-matched development pathway using paired tissue and plasma sampling, relevant resistant models, causal perturbation, and pharmacodynamic validation is proposed. Clinical decisions should follow validated mechanisms rather than extrapolated biophysical hypotheses.

## 1. Introduction

Based on GLOBOCAN 2024 estimates published in July 2026, lung cancer was the most frequently diagnosed cancer worldwide, with approximately 2.6 million new cases (12.8% of all cancers), and the leading cause of cancer death, with approximately 1.9 million deaths (19.1% of cancer deaths) in 2024 [1]. Non-small cell lung cancer (NSCLC) accounts for approximately 80–85% of lung cancers [2,3]. Activating EGFR mutations defines one of the most clinically actionable molecular subsets. Their prevalence is not uniform: it is highest in lung adenocarcinoma, never-smokers, women, and East Asian populations, with broad geographic estimates ranging from approximately 10–15% in many Western cohorts to 30–50% in selected Asian cohorts [4,5]. The clinical impact of these mutations is disproportionately large because EGFR-mutant tumors can remain oncogene-dependent through several lines of therapy, even while acquiring complex resistance mechanisms.

The 2004 reports linking exon 19 deletions and the L858R substitution to dramatic gefitinib responses converted a clinical observation into a molecular treatment paradigm [6,7,8]. Randomized trials subsequently established first- and second-generation EGFR tyrosine kinase inhibitors (TKIs) as superior to platinum-doublet chemotherapy for progression-free survival (PFS) in mutation-selected disease [9,10,11,12,13,14,15,16,17]. The T790M gatekeeper mutation then exposed the central evolutionary limitation of reversible ATP-competitive inhibition. Osimertinib, a mutant-selective covalent inhibitor with central nervous system (CNS) activity, overcame T790M and later displaced earlier TKIs in the first-line setting [18,19,20,21]. It has since expanded into resected disease, unresectable stage III disease after chemoradiotherapy, and intensified first-line regimens [22,23,24,25,26].

These advances have not removed the problem of resistance; they have changed its architecture. Under first-generation inhibition, one lesion—T790M—accounted for approximately one-half of acquired resistance. Under first-line osimertinib, no single lesion dominates. MET amplification, EGFR C797S, HER2 amplification, RAS-MAPK activation, PI3K-pathway lesions, oncogenic fusions, epithelial-mesenchymal transition (EMT), small-cell or squamous transformation, and non-genetic persistence can coexist within the same patient [27,28,29,30,31,32,33,34,35,36,37,38,39,40,41,42,43,44,45,46,47,48,49,50,51,52,53,54,55,56,57,58]. This heterogeneity undermines the assumption that an increasingly potent kinase inhibitor can, by itself, eradicate every resistant state.

Genomics identifies altered signaling components, but it does not fully describe the pharmacological state in which signaling persists. Membrane potential, calcium entry, pH, cell volume, receptor trafficking, organellar function, apoptosis, and stromal growth-factor supply can determine whether pathway inhibition produces durable cell death or a reversible tolerant state [59,60,61,62,63,64,65]. These variables are controlled by ion channels and transporters, but also by adaptive kinase hubs, lineage regulators, autophagy, and anti-apoptotic proteins. They should therefore be evaluated as a compensatory network rather than as isolated molecular targets while keeping in mind that network membership is not the same as demonstrated dependency; the distinction is defined operationally in Section 7 and applied throughout.

This review has three objectives. First, it updates the clinical pharmacology and molecular architecture of EGFR resistance through June 2026. Second, it ranks compensatory targets according to clinical evidence, causal perturbation, target engagement, exposure compatibility, and safety, using an evidence framework that reports clinical and mechanistic maturity separately rather than merging them into a single score. Third, it places ion-channel and transporter programs within a broader pharmacological hierarchy that includes on-target EGFR mutations, RTK bypass, downstream convergence, lineage plasticity, drug-tolerant persistence, and ADC payload delivery. The intended contribution is not another catalogue of resistance mechanisms. Previous reviews have treated EGFR resistance genomics and cancer ion-channel biology as separate literatures; here, they are placed on one explicit evidence scale so that the reader can see precisely where a biophysical program stands relative to a clinically validated bypass mechanism, and what experiment would be required to move it. The biological rationale-linking membrane potential, calcium signaling, pH regulation, and cell volume to drug-tolerant persistence is plausible and is developed in Section 7; it should be read as a rationale for investigation, not as evidence of a causal role in osimertinib resistance, for which no Level A or Level B human data currently exist.

## 2. Literature Search Strategy and Evidence-Grading Framework

A structured narrative search of PubMed/MEDLINE was conducted and iteratively updated through 15 June 2026, with no start-date limit so that foundational pharmacology could be retrieved. Modular searches combined terms for: (i) EGFR-mutant NSCLC and first-, second-, third-, or fourth-generation EGFR TKIs; (ii) acquired resistance, C797S, MET, HER2/HER3, TROP2, RAS-MAPK, PI3K-AKT, oncogenic fusions, EMT, histologic transformation, and drug-tolerant persistence; (iii) bispecific antibodies, antibody-drug conjugates, liquid biopsy, minimal residual disease, organoids, patient-derived xenografts, single-cell sequencing, and spatial omics; and (iv) ANO1/TMEM16A, CLIC1, CLCA, LRRC8/VRAC, KCNN4/KCa3.1, KCNH2/hERG, STIM/ORAI, TRP channels, SCN5A/Nav1.5, SCN9A/Nav1.7, NHE1, and HVCN1. The principal Boolean strings for each module are reproduced in Appendix A. Searches were supplemented by backward and forward citation tracking and by hand-searching the 2023–2026 proceedings of ASCO, ESMO, AACR, and the World Conference on Lung Cancer. Selection prioritized prospective trials, paired human specimens, longitudinal ctDNA, patient-derived models, causal perturbation studies, and foundational pharmacology. Source types were handled hierarchically: peer-reviewed primary reports were cited for all quantitative and mechanistic claims wherever one existed; conference abstracts were cited only where no full publication had appeared and are identified as abstracts in the reference list and at the point of use; regulatory decisions were taken from the issuing agency or the sponsor’s dated announcement; and review articles were used to identify the primary literature and are not cited as the sole support for any specific quantitative or mechanistic statement. For the epidemiological background alone, the subsequently published GLOBOCAN 2024 estimates (released in July 2026) were additionally checked in October 2026; this update does not extend the 15 June 2026, search cutoff applied to EGFR-resistance pharmacology.

Because the topic spans clinical oncology, evolutionary biology, molecular pharmacology, electrophysiology, and drug development, this manuscript is a structured narrative review rather than a formal systematic review or meta-analysis. Heterogeneous efficacy estimates were not pooled, and a single risk-of-bias instrument was not applied across incompatible study designs. The evidence levels used throughout grade one thing only: the strength of the evidence that a given molecular event or target is operative in acquired resistance in EGFR-mutant NSCLC and can be addressed therapeutically in that setting. They do not grade the methodological quality of an individual publication, and they are not a measure of a drug’s overall development maturity. To remove the ambiguity created by placing clinical and experimental evidence at the same level, the two domains are kept separate:

Level A: randomized or prospective clinical validation in EGFR-mutant NSCLC. A mechanism-matched intervention improved a clinical endpoint in a randomized trial conducted in this population, or the mechanism was prospectively validated as a treatment-selection biomarker in a registration-directed trial. Level A does not by itself establish molecular causality.

Level B: non-randomized human evidence in EGFR-mutant NSCLC. The mechanism is reproducibly detected in patients and linked to treatment response or outcome: single-arm prospective trials, paired pre-treatment and progression specimens, longitudinal ctDNA series, and retrospective multi-institutional cohorts. Level B establishes that a mechanism occurs in patients, not that targeting it changes outcome.

Level C: experimental causal evidence generated in EGFR-mutant NSCLC systems. Genetic or pharmacological perturbation in isogenic EGFR-mutant resistant lines, patient-derived organoids, or patient-derived xenografts established from EGFR-mutant NSCLC. Level C can establish causality within a model but says nothing about human relevance or clinically achievable exposure.

Level D: indirect evidence. This includes mechanistic data from other tumor types, from EGFR-wild-type NSCLC, or from normal physiology. Level D supports biological plausibility only and does not establish that the mechanism operates in EGFR-mutant NSCLC.

Two consequences follow, and both are applied consistently in the tables. First, the levels are not strictly ordinal across domains. A Level C claim supported by genetic loss-of-function with rescue and by orthogonal chemotypes may be mechanistically more secure than a Level B association, yet only Level A evidence supports a treatment recommendation. Where clinical and experimental maturity diverge, both are reported in the form “Level B clinical/Level C mechanistic”, and clinical actionability must be evaluated from intervention-specific human evidence rather than inferred from a mechanistic grade. Second, a mechanism can be clinically validated without being mechanistically understood, and mechanistically secure without being clinically useful; this review states which of the two is missing rather than averaging them into a single grade. Evidence derived directly in EGFR-mutant NSCLC is never assigned the same level as evidence extrapolated from another tumor type: the latter is capped at Level D irrespective of the rigor of the original study.

Pharmacological claims were judged against five criteria that are applied independently of the A-D levels: demonstrated target engagement at the intended site of action; free (unbound) drug exposure above the effective concentration for a relevant duration; interpretable concentration-response behavior; concordance across orthogonal chemotypes and genetic perturbation, including rescue; and an acceptable therapeutic index. These criteria constrain whether a target at any evidence level can be pursued clinically; satisfying them does not raise a target’s evidence level, and failing them does not lower it. A target that is Level A by clinical validation may still fail the exposure criterion in a new combination, and a Level C target may satisfy all five pharmacological criteria and still lack any human evidence.

Quantitative values were extracted preferentially from primary reports. Apparent discrepancies were retained rather than harmonized when they arose from different treatment lines, assay platforms, or biopsy compartments. Amplification frequencies detected by plasma sequencing, for example, are expected to be lower than those found by tissue next-generation sequencing (NGS) or fluorescence in situ hybridization (FISH), while histologic transformation cannot be diagnosed from plasma alone. Figure 1 provides the visual roadmap for the review by linking landmark EGFR-directed therapeutic eras to the parallel expansion of residual-state diversity.

## 3. Pharmacological Evolution of EGFR-Directed Therapy

### 3.1. From Reversible ATP Competition to Mutant-Selective Covalent Inhibition

IPASS provided the decisive demonstration that the clinical phenotype is an imperfect surrogate for genotype. In the EGFR-mutant subgroup, gefitinib produced a marked PFS advantage over carboplatin-paclitaxel, whereas EGFR-wild-type tumors did worse with gefitinib [9]. Subsequent mutation-selected trials consistently showed higher objective response rates (ORRs) and longer PFS with gefitinib, erlotinib, or afatinib than with chemotherapy [10,11,12,13,14,15,16,17]. These trials established oncogene addiction as a therapeutic principle, but median PFS remained approximately 9–14 months and CNS progression was frequent. For a compound-by-compound summary of primary targets and pharmacological mechanisms, see Appendix B (Table A1).

The biochemical explanation for the most common acquired lesion was refined over several years. T790M does not merely create steric hindrance; it restores high ATP affinity to the mutant kinase, reducing the competitive advantage of first-generation inhibitors [66,67,68,69]. Osimertinib was designed to bind covalently to Cys797 while sparing wild-type EGFR to a clinically useful degree. In AURA3, osimertinib improved median PFS from 4.4 to 10.1 months compared with platinum-pemetrexed in T790M-positive disease (hazard ratio [HR], 0.30) [19]. In untreated disease, FLAURA improved median PFS from 10.2 to 18.9 months (HR, 0.46) and median overall survival (OS) from 31.8 to 38.6 months (HR, 0.80) compared with gefitinib or erlotinib [20,21]. These results shifted the dominant resistance question from how to treat T790M to how to manage a genomically and phenotypically diverse post-osimertinib state.

### 3.2. Earlier-Stage Exposure and First-Line Pharmacological Intensification

The therapeutic footprint of osimertinib now extends beyond metastatic monotherapy. In ADAURA, adjuvant osimertinib produced a profound disease-free survival (DFS) benefit after complete resection, and the mature analysis showed an OS benefit: 5-year OS was 88% with osimertinib and 78% with placebo in the overall stage IB-IIIA population (HR for death, 0.49; 95% CI, 0.34–0.70) [22,23]. In LAURA, consolidation osimertinib after definitive chemoradiotherapy for unresectable stage III EGFR-mutant NSCLC improved median PFS from 5.6 to 39.1 months (HR, 0.16) [25]. These studies reduce recurrence risk but also create new biological contexts in which resistance may emerge from microscopic residual disease rather than bulky metastatic tumors.

Two first-line intensification strategies have altered the metastatic landscape. FLAURA2 added platinum-pemetrexed to osimertinib and improved investigator-assessed median PFS from 16.7 to 25.5 months (HR, 0.62), with a larger absolute benefit in several high-risk subgroups but greater hematologic toxicity [24]. In the final analysis, adding chemotherapy also improved median overall survival from 37.6 to 47.5 months (HR, 0.77; 95% CI, 0.61–0.96; *p* = 0.02), with 3-year overall survival of 63% versus 51%: the longest overall survival reported in a first-line EGFR-mutant phase III trial to date [70]. MARIPOSA combined the EGFR-MET bispecific antibody amivantamab with the third-generation TKI lazertinib and improved blinded independent central review PFS over osimertinib [26]. In the final overall survival analysis, amivantamab–lazertinib significantly prolonged survival (median not reached versus 36.7 months; HR, 0.75; *p* = 0.005; 3-year overall survival 60% versus 51%) at the cost of higher grade 3 or higher toxicity (80% versus 52%) [71]. Regional third-generation agents, including furmonertinib, aumolertinib, and lazertinib, also demonstrate that CNS penetration, mutant selectivity, dose, and regional development strategy influence class performance [72,73,74]. Earlier antiangiogenic intensification with ramucirumab–erlotinib or bevacizumab–erlotinib provided additional proof that non-EGFR pharmacology can delay progression, although toxicity and sequencing remain relevant [75,76].

For EGFR exon 20 insertion disease, the PAPILLON trial demonstrated that amivantamab plus carboplatin–pemetrexed improved median PFS from 6.7 to 11.4 months (HR, 0.40; 95% CI, 0.30–0.53) [77]. New exon 20-selective TKIs, including sunvozertinib, have also produced clinically meaningful activity after platinum therapy [78,79]. Mobocertinib provided structural and clinical proof of concept but also illustrated the narrow wild-type EGFR therapeutic index of this mutation class [80,81]. These uncommon variants should not be conflated with classical exon 19 deletion/L858R resistance biology because their kinase conformations and pharmacological windows differ [82].

Table 1 lists the landmark trials used as quantitative anchors for the evolution of EGFR-directed pharmacology.

## 4. Pharmacological Architecture of Resistance

Resistance should be conceptualized as a topology of coexisting routes rather than a mutually exclusive list. A single patient may carry a plasma-detectable C797S clone, tissue-level MET amplification, and a spatially separate transformed lesion. Treatment line matters: later-line osimertinib is often applied to a T790M-positive population and therefore generates compound-allele problems, whereas first-line osimertinib resistance usually develops without T790M. Sampling matters equally. Plasma ctDNA is highly informative for single-nucleotide variants and clonal dynamics, but it has lower sensitivity for copy-number gains and cannot diagnose SCLC or squamous transformation [36,37,38,39,57,84,85,86,87,88,89,90,91,92]. Figure 2 translates the resistance architecture into an assay-aware atlas, emphasizing that plasma, tissue, and functional models answer different pharmacological questions.

### 4.1. On-Target EGFR Alterations: C797S and Beyond

C797S replaces the nucleophilic cysteine required for osimertinib’s covalent bond and is the best characterized on-target resistance event [33]. Its therapeutic meaning depends on allelic context. When T790M and C797S are in trans, combined first- and third-generation TKIs can, in principle, inhibit separate alleles; when they are in cis, the same combination is ineffective [34]. This distinction is clinically relevant mainly after later-line osimertinib because first-line resistance generally lacks T790M. Other EGFR alterations, including L718Q/V, G724S, L792 substitutions, exon 20 insertions, and EGFR amplification, alter inhibitor binding or kinase conformation and show mutation-specific sensitivity patterns [39,82,93,94,95].

Fourth-generation programs have pursued ATP-competitive mutant selectivity, allosteric binding, or dual-site inhibition. EAI045 demonstrated that an allosteric pocket could be exploited to inhibit L858R/T790M/C797S, but the compound required cetuximab in relevant models because receptor dimerization limited access [96]. JBJ-04-125-02 and related allosteric molecules showed that combining an allosteric inhibitor with osimertinib can deepen suppression and delay resistance in preclinical systems [97]. Brigatinib plus an anti-EGFR antibody, CH7233163, BLU-945, and BBT-176 provide additional proof of concept [93,94,95,98,99]. However, early molecular responses in dose-escalation studies do not establish a new standard. Mutation spectrum, CNS exposure, wild-type EGFR selectivity, and tolerability of antibody combinations remain decisive development constraints.

### 4.2. MET, ERBB2/HER3, RAS-MAPK, PI3K, and Oncogenic Fusions

MET amplification is the most reproducible bypass mechanism after osimertinib. The canonical mechanism is ERBB3-dependent reactivation of PI3K-AKT signaling despite EGFR inhibition [27,28]. Reported frequencies are typically approximately 10–20%, but the estimate varies with treatment line and assay. Plasma NGS underestimates focal amplification; tissue NGS, FISH, and immunohistochemistry measure related but non-identical biological states. HGF-mediated MET activation can also produce resistance without high-level amplification [29]. These observations explain why the clinically useful biomarker threshold for an EGFR-MET combination remains a central question.

ERBB2 amplification, PIK3CA mutations, BRAF or KRAS alterations, and acquired RET, ALK, BRAF, or other fusions occur less frequently but are collectively important [30,36,37,38,39]. Many are subclonal and may be detectable only in one compartment. HER3 is mechanistically distinct: it has weak intrinsic kinase activity but functions as a signaling hub and is broadly expressed in EGFR-mutant tumors. Its role as an ADC target does not require HER3 to be the genomic resistance driver. This separates target abundance and payload delivery from pathway addiction, a principle that also applies to TROP2-directed ADCs. These alterations should not be treated as a single actionable category. MET amplification above a defined threshold, ERBB2 amplification, and RET or ALK fusions are potentially actionable because a drug matched to each exists and has produced responses in biomarker-selected patients. PIK3CA mutations, low-level copy-number gains, and many co-occurring RAS-pathway events are of uncertain functional relevance: they are frequently subclonal, are often detected alongside a dominant mechanism, and have not been shown to confer dependency when targeted. Clonality, allele fraction relative to the founding EGFR mutation, and whether the alteration is present in tissue, as well as plasma, are the practical discriminators, and none of them is settled by detection alone.

### 4.3. Histologic Transformation and Lineage Plasticity

Transformation to SCLC is a high-consequence resistance state that cannot be diagnosed by ctDNA alone. Retrospective series report SCLC transformation in approximately 5–15% of resistant cases, with higher risk in tumors carrying concurrent RB1 and TP53 loss [51,52,53,54]. Transformed tumors usually retain the original EGFR mutation, supporting clonal continuity, but lose EGFR dependence and adopt neuroendocrine transcriptional programs. Platinum–etoposide is commonly used, yet outcomes remain inferior to de novo SCLC. Squamous transformation and EMT are additional manifestations of lineage plasticity [38,49,55,56]. These states demonstrate that resistance can arise by changing cell identity rather than merely changing a drug-binding residue.

The literature is not fully concordant on whether transformation is induced by treatment or selected from a pre-existing lineage-competent clone. Paired genomic studies support both models. RB1/TP53-deficient tumors may possess an intrinsic capacity for neuroendocrine transition, while treatment pressure and epigenetic remodeling determine when that capacity becomes phenotypically dominant [52,53,54,55,56]. A balanced interpretation is therefore that the genotype creates competence, whereas therapy and microenvironment shape state transition.

### 4.4. Non-Genetic Tolerance, EMT, and Microenvironmental Protection

Drug-tolerant persisters are rare cells that survive otherwise lethal pathway inhibition without a fixed resistance mutation. Reversible chromatin remodeling, altered apoptotic priming, AXL activation, YAP/TEAD signaling, oxidative stress responses, autophagy, and metabolic rewiring have all been implicated [40,41,42,43,44,45,46,47,48,49,50]. Hata and colleagues showed that resistant colonies can arise either by selection of pre-existing clones or through evolution from drug-tolerant cells, establishing two distinct temporal paths [40]. Single-cell studies of patient tumors further demonstrated treatment-induced shifts in epithelial state, immune interactions, and bypass pathways [45].

This non-genetic reservoir is relevant to the channelome because membrane potential, Ca^2+^ signaling, pH regulation, and volume adaptation are state variables that can change rapidly without mutation. The key unresolved issue is causality. A channel may be required to maintain the persister state, merely co-expressed with that state, or induced as a consequence of altered lineage. These alternatives require perturbation and rescue experiments in longitudinal EGFR-mutant models, not transcriptomic correlation alone.

Table 2 summarizes the principal resistance mechanisms, approximate frequencies, preferred detection methods, and caveats that explain discordant estimates across cohorts. A consolidated treatment-context matrix is provided later in the Discussion to integrate the sampling compartment, mechanism-matched action, and evidence supporting each resistance state.

## 5. Clinically Actionable Pharmacological Strategies After Osimertinib

### 5.1. Re-Biopsy, Progression Pattern, and Pharmacological Triage

The first therapeutic decision is diagnostic. Oligoprogression, isolated CNS progression, and systemic progression should not be treated as the same state. Local ablative therapy with continued osimertinib can be reasonable for limited progression when most disease remains controlled. Systemic progression should trigger plasma ctDNA and, whenever feasible, tissue biopsy. Tissue is particularly important when plasma is negative, copy-number change is suspected, or transformation is clinically plausible. A negative plasma result is not evidence of absence; low tumor shedding and CNS-confined disease can produce false-negative ctDNA. The resulting allocation is consolidated later in the Discussion, with the sampling compartment, mechanism-matched action, and evidence level specified for each resistance state.

### 5.2. MET-Directed Combinations and EGFR-MET Bispecific Pharmacology

Dual EGFR-MET inhibition has a strong mechanistic basis in MET-amplified resistance. In the final analysis of the phase Ib TATTON study, osimertinib plus savolitinib produced objective response rates of 33% to 67% across the Part B expansion cohorts and 62% in Part D; the lowest rate was observed in patients previously treated with a third-generation TKI (Part B1) and the highest in third-generation-TKI-naive cohorts, and the response also varied with the assay used to define MET positivity [100,101]. INSIGHT 2 reported clinically meaningful activity for tepotinib plus osimertinib in MET-amplified tumors after first-line osimertinib [102]. The activity of crizotinib, capmatinib, and tepotinib in MET exon 14-altered or MET-amplified NSCLC confirms that MET is pharmacologically tractable, although those disease settings are not interchangeable with acquired EGFR-TKI resistance [103,104,105]. Cross-trial comparison remains difficult because MET eligibility has been defined by FISH, NGS copy number, and/or immunohistochemistry. Prospective harmonization of MET thresholds is needed to avoid both under-selection and treatment of non-addicted, low-level gain. The distinct molecular targets of the agents discussed here and below are also summarized in Table A1.

Amivantamab combines EGFR and MET binding with receptor downregulation and immune-effector functions. In platinum-pretreated EGFR exon 20 insertion disease, CHRYSALIS showed an ORR of 40%, median duration of response of 11.1 months, and median PFS of 8.3 months [106]. After progression on osimertinib, MARIPOSA-2 demonstrated that amivantamab plus carboplatin-pemetrexed improved PFS and ORR compared with chemotherapy alone; the triplet, including lazertinib, also improved PFS but increased hematologic toxicity [107]. MET-directed ADC pharmacology is also being explored; telisotuzumab vedotin has shown activity in c-MET protein-overexpressing NSCLC, but target definition and applicability to EGFR-resistant disease remain distinct questions [108].

### 5.3. ADC Pharmacology: HER3, TROP2, and Payload Dependence

ADCs bypass the requirement for a single dominant kinase driver by using surface antigens to deliver a cytotoxic payload. Patritumab deruxtecan (HER3-DXd) links a HER3-directed antibody to a topoisomerase I inhibitor. In the phase I dose-expansion cohort treated at 5.6 mg/kg (*n* = 57), confirmed ORR by blinded independent central review (BICR) was 39% (95% CI, 26.0–52.4), and the median PFS was 8.2 months (95% CI, 4.4–8.3) across diverse resistance mechanisms [109]. In the single-arm phase II HERTHENA-Lung01 trial (*n* = 225), after EGFR TKI and platinum therapy, confirmed ORR was 29.8% (95% CI, 23.9–36.2), median PFS was 5.5 months, and median OS was 11.9 months [110]. Both are non-randomized studies, and their response rates cannot be read as comparable to randomized outcomes. Activity across heterogeneous genomic subgroups supports payload delivery as the principal therapeutic logic, although interstitial lung disease (ILD), cytopenias, and the absence of a validated predictive HER3 threshold remain important. The confirmatory randomized phase III HERTHENA-Lung02 trial, whose design was published separately [111], compared patritumab deruxtecan with platinum–pemetrexed after progression on a third-generation EGFR TKI. Its efficacy results have so far been reported only in congress abstract form [112]: the primary endpoint of BICR-assessed progression-free survival was met (median 5.8 versus 5.4 months; HR, 0.77; 95% CI, 0.63–0.94; *p* = 0.011) and confirmed ORR was 35.2% (95% CI, 29.7–40.9) versus 25.3% (95% CI, 20.4–30.6), while overall survival data were immature at the protocol-specified interim analysis. On 29 May 2025, the sponsors voluntarily withdrew the U.S. Biologics License Application, which had sought accelerated approval on the strength of the single-arm HERTHENA-Lung01 trial [113]. Because these outcomes are currently reported in a meeting abstract, the PFS and ORR estimates remain provisional pending peer-reviewed full reporting; the cited abstract does not provide mature OS estimates, and regulatory status is jurisdiction- and time-specific and was last verified on 17 October 2026. These findings do not establish an overall survival advantage or a predictive HER3-expression threshold: the antigen functions as a delivery address rather than as evidence of pathway dependence.

Datopotamab deruxtecan (Dato-DXd) targets TROP2 and also delivers a topoisomerase I payload. The single-arm phase II TROPION-Lung05 study demonstrated activity in heavily pretreated NSCLC with actionable genomic alterations, including EGFR mutations [114]. In the randomized phase III TROPION-Lung01, Dato-DXd significantly improved the co-primary endpoint of progression-free survival versus docetaxel in the overall population (4.4 versus 3.7 months; HR, 0.75; 95% CI, 0.62–0.91; *p* = 0.004); the benefit was driven almost entirely by non-squamous histology (5.5 versus 3.6 months; HR, 0.63; 95% CI, 0.51–0.79), whereas squamous histology showed a numerically unfavorable hazard ratio, and the co-primary overall survival endpoint was not met (12.9 versus 11.8 months; HR, 0.94; 95% CI, 0.78–1.14; *p* = 0.53) [115]. A regulatory pooled analysis of the EGFR-mutant patients from these two trials (*n* = 114) reported a confirmed ORR of 45% (95% CI, 35–54) and a median duration of response of 6.5 months (95% CI, 4.2–8.4), and supported accelerated approval in the United States on 23 June 2025, for EGFR-mutated NSCLC after prior EGFR-directed therapy and platinum-based chemotherapy; this approval was still in force when regulatory status was last verified on 17 October 2026 [116]. A separately published pooled analysis of the same two trials defined a slightly different population (*n* = 117) and reported an ORR of 43% and a median duration of response of 7.0 months [117]; the two analyses are not interchangeable, and the regulatory figures are used here because they define the approved indication. Interstitial lung disease, stomatitis, and a narrow therapeutic index further constrain ADC combination development. Trastuzumab deruxtecan has separately demonstrated the feasibility of HER2-directed payload delivery in HER2-mutant NSCLC [118]. These studies illustrate that a biomarker-defined population may benefit even when a broader trial endpoint or a different antigen-defined subgroup is less informative, and that single-arm response rates must not be read as comparable to randomized outcomes.

ADC selection should not be reduced to target immunohistochemistry alone. Internalization, linker cleavage, payload sensitivity, bystander effect, drug efflux, and DNA-damage response all influence efficacy. Cross-resistance between deruxtecan-containing ADCs is not yet fully defined. Sequential use may select shared payload resistance even when the surface antigen changes.

### 5.4. Immunotherapy: Limited Single-Agent Activity and Combination Caution

EGFR-mutant NSCLC generally derives less benefit from single-agent PD-1/PD-L1 blockade than smoking-associated, EGFR-wild-type disease, even when PD-L1 is expressed [119,120]. Low tumor mutational burden, an immune-excluded microenvironment, and oncogene-driven PD-L1 regulation contribute. Combinations of immunotherapy, chemotherapy, and antiangiogenic therapy can produce activity after TKI failure, as suggested by the EGFR-mutant subgroup of IMpower150 [121], but toxicity, prior TKI exposure, and sequence-dependent pneumonitis require caution. Immunotherapy should therefore not displace a targetable resistance mechanism or a proven post-osimertinib regimen. The evidence supports no stronger statement than this. The IMpower150 signal comes from a subgroup of a trial not designed to answer this question, in patients treated before current first-line standards; it does not establish a sequencing rule, and it cannot be extended to patients progressing on first-line osimertinib or on amivantamab-lazertinib, in whom the regimen has not been tested. Conclusions about checkpoint-inhibitor efficacy in EGFR-mutant disease should be confined to the specific population, line of therapy, and combination in which they were observed.

Table 3 compares selected post-osimertinib pharmacological strategies with their efficacy signals and development limitations.

## 6. Pharmacological Integration of Compensatory Networks Beyond EGFR

Resistance targets should be classified as dominant drivers, convergent signaling hubs, permissive state regulators, delivery antigens, or biophysical modifiers because these categories imply different pharmacological strategies. MET amplification is the clearest clinically actionable receptor-level bypass, whereas HER3 and TROP2 often function primarily as ADC delivery addresses rather than obligate resistance drivers. AXL, IGF1R, FGFR, SRC/FAK, SHP2-RAS-MAPK, PI3K-AKT-mTOR, YAP/TEAD, and JAK/STAT programs can maintain survival or facilitate the transition from reversible persistence to fixed resistance [27,28,29,30,40,41,42,43,44,45,46,47,48,49,50,122,123,124].

Drug-development priorities differ across these classes. On-target EGFR alterations require mutant selectivity, allelic phasing, CNS penetration, and suppression at tolerable wild-type EGFR exposure. RTK bypass requires proof that the amplified or ligand-activated pathway is functionally dominant rather than a passenger copy-number event. Convergent signaling hubs such as SHP2 or MEK may broaden coverage but can be limited by adaptive feedback, normal-tissue toxicity, and a narrow combination window [122,123]. ADC pharmacology depends on antigen density, internalization, linker behavior, bystander effect, payload sensitivity, efflux, and DNA-damage response rather than antigen expression alone.

Cell-state and lineage mechanisms require a different logic. Drug-tolerant persisters, EMT, AXL-high states, autophagy, lysosomal adaptation, and altered BCL-2-family dependence are reversible or context-dependent and therefore need serial pharmacodynamic measurements rather than a single baseline genotype [40,41,42,43,44,45,46,47,48,49,124]. Small-cell or squamous transformation requires histology-specific therapy rather than escalation of EGFR inhibition. Ion channels and transporters constitute one emerging modifier class within this network. ANO1 is a mechanistically instructive example of receptor coupling and membrane organization, but no channel target should be promoted as a general resistance breaker until dependency, exposure, and safety are demonstrated in longitudinal EGFR-mutant patient-derived models. Figure 3 summarizes the compensatory pharmacology that extends beyond EGFR and places each target class behind a biomarker, exposure, target-engagement, and safety gate. Across all five classes, the same evidential standard applies: membership of the compensatory network is a reason to investigate a target, not evidence that the tumor depends on it. The operational definition that separates an association from a candidate target and from a demonstrated dependency is given at the head of Section 7 and is applied throughout the subsequent target-specific and summary tables.

## 7. Ion Channels and Transporters as Candidate Pharmacological Targets: Evidence Status and Translational Requirements

Ion channels and transporters occupy the lowest evidence tier of the resistance network considered in this review. They are pharmacologically attractive because they regulate membrane potential, calcium entry, pH, cell volume, receptor trafficking, lysosomal function, migration, and apoptotic competence on timescales that are not captured by DNA sequencing alone [60,61,62,63,64,65]. These systems may contribute to reversible tolerance or amplify a genetically defined bypass, but channel expression should not be equated with channel dependency.

Because that distinction is central to the argument of this section, the terminology is defined explicitly and used consistently from this point onward. A dependency is claimed only where genetic loss-of-function reduces the resistance phenotype, the effect is reversed by re-expression of the wild-type protein, and at least one structurally unrelated pharmacological agent reproduces the loss-of-function phenotype at an exposure compatible with the measured target engagement. A candidate target denotes a protein for which perturbation alters a cancer-relevant phenotype but for which one or more of those three conditions has not been met. An association denotes expression-, copy-number-, or survival-level correlation only. By this definition, no ion channel or transporter discussed below currently qualifies as a dependency in acquired resistance to osimertinib in EGFR-mutant NSCLC; all are candidate targets or associations, which is why the section title and Figure 4 use that language. Accordingly, the word “dependency” is used in this section only to name the question under investigation, never as a description of the current evidence for any individual target.

The central translational question is therefore dependency, not expression. A credible oncochannel target must satisfy the definition above and, in addition, the five pharmacological criteria set out in Section 2, of which three are particularly unforgiving for this target class: concentration-response behavior must be interpretable at free-drug exposures compatible with human dosing; target engagement must be demonstrated directly, for an ion channel most convincingly as suppression of the relevant current rather than as a change in viability; and the rescue construct must, where appropriate, distinguish conductance from scaffolding. Many historical channel inhibitors are promiscuous at micromolar concentrations, and systemic on-target physiology can eliminate an otherwise attractive therapeutic window. Figure 4 narrows the focus to ion-channel and transporter circuits, explicitly treating these targets as candidate targets under investigation rather than as demonstrated dependencies or clinically validated assignment tools.

### 7.1. Chloride-Conducting Systems: ANO1, CLIC1, CLCA, and VRAC

ANO1/TMEM16A is a Ca^2+^-activated chloride channel encoded at 11q13, a region frequently amplified in epithelial cancers. The identification of TMEM16A as a channel-forming protein was independently reported in 2008 [125,126,127], and structural studies subsequently clarified its Ca^2+^-dependent gating architecture and the mechanistic diversity of TMEM16 channel/scramblase family members [128,129]. In cancer models, ANO1 can support MAPK signaling, proliferation, migration, and tumor growth [130,131,132]. ANO1 and EGFR can exist in a signaling complex in head-and-neck cancer models [132], while ANO1 promoted EGFR and CAMK signaling in breast cancer models [131]. These data establish biochemical plausibility, not disease-specific proof of osimertinib resistance in lung cancer.

Ani9 inhibits ANO1 with submicromolar-to-nanomolar potency in heterologous systems and has substantially less activity against ANO2 [133]. T16Ainh-A01, CaCCinh-A01, niclosamide, and several natural-product scaffolds have also been used, but their selectivity profiles differ [133,134]. The translational challenge is to separate channel conductance from scaffold function. If ANO1 stabilizes an EGFR-containing membrane complex, a pore blocker may not reproduce genetic depletion. Conversely, a conductance-deficient rescue construct can test whether ion flux is necessary. This distinction has not been resolved in a paired pre/post-osimertinib NSCLC system.

CLIC1 is a metamorphic protein that can exist in soluble and membrane-associated states. It has been linked to redox biology, cell-cycle progression, migration, and stem-like tumor populations [135,136]. However, the name “chloride intracellular channel” can be misleading because several reported functions are enzymatic or scaffolding rather than canonical pore conductance. Evidence in lung cancer is mainly expression-based or derived from conventional cell lines; direct linkage to EGFR-TKI resistance is weak. CLCA family proteins are best treated as regulators of chloride conductance and epithelial differentiation rather than simple ion channels; CLCA2 can be induced by p53 and suppress proliferation [137].

Volume-regulated anion channels (VRACs), assembled from LRRC8 heteromers, provide a stronger pharmacological bridge to chemotherapy. LRRC8 composition determines transport of organic osmolytes and contributes to cellular uptake or response to platinum drugs [138,139,140]. Because platinum–pemetrexed is now used both in first-line intensification and after osimertinib, the LRRC8 state may influence regimen sensitivity independently of EGFR. This is a testable biomarker hypothesis, but it should not be confused with an established mechanism of osimertinib resistance.

### 7.2. Potassium Channels: KCa3.1 and hERG

KCa3.1, encoded by KCNN4, is a voltage-independent, Ca^2+^/calmodulin-activated K^+^ channel. Its opening hyperpolarizes the plasma membrane and thereby preserves the electrochemical driving force for sustained Ca^2+^ entry. This creates a functional circuit between KCa3.1 and store-operated Ca^2+^ entry. TRAM-34 is a potent experimental KCa3.1 blocker [141,142], while senicapoc has prior human exposure in sickle cell disease [143]. General potassium-channel and cancer literature supports roles in proliferation, migration, and immune-cell function [62,63,64,144,145]. In EGFR-mutant NSCLC, however, paired expression after osimertinib, genetic rescue, and synergy at clinically achievable exposure remain insufficiently developed. Senicapoc should therefore be described as a repurposing candidate, not an established lung-cancer therapy.

hERG/KCNH2 channels have been associated with proliferation, survival, adhesion signaling, and cancer biomarkers [144,145,146,147,148]. Their therapeutic interpretation is unusually difficult because hERG is a central cardiac repolarization channel. Systemic block can prolong QT and provoke torsades de pointes [147]. Many anticancer molecules inhibit hERG unintentionally, so reduced tumor growth after a nominal hERG blocker may coexist with unacceptable cardiac risk and extensive off-target pharmacology. A viable cancer strategy would require tumor-selective delivery, exploitation of a cancer-specific hERG complex, or modulation of non-conducting scaffold functions without blocking cardiac IKr.

### 7.3. Calcium Entry: STIM-ORAI and TRP Channels

Store-operated calcium entry (SOCE) is initiated when endoplasmic-reticulum Ca^2+^ depletion causes STIM proteins to cluster and engage ORAI channels at the plasma membrane [149,150,151,152,153]. In tumor models, STIM1-ORAI1 signaling regulates migration, focal adhesion turnover, NFAT activation, proliferation, and metastasis, while ORAI3 can support hormone- and AKT-linked tumorigenic programs in selected cancers [154,155,156]. In NSCLC, dysregulated ORAI/STIM expression and Ca^2+^ entry have been implicated in growth and chemotherapy resistance, but model dependence is substantial. The strongest mechanistic design would combine patch clamp or Ca^2+^ imaging with CRISPR loss-of-function, rescue, phosphoproteomics, and osimertinib dose response in EGFR-mutant parental and resistant pairs.

TRP channels are polymodal sensors that conduct Ca^2+^, Na^+^, and other cations in response to lipids, temperature, mechanical stress, redox state, or receptor signaling [157,158,159,160]. TRPC, TRPM, and TRPV family members have been associated with cancer-cell proliferation, migration, invasion, and therapy response, but their effects are not interchangeable. TRPM7, for example, combines channel and kinase domains and has been required for metastasis in breast-cancer models [159]. A review of EGFR resistance should resist collapsing all TRP channels into a single pro-oncogenic category. Expression direction, subcellular localization, ion selectivity, and the relevant agonist environment determine phenotype.

### 7.4. Sodium and Proton Transport: Nav1.5/Nav1.7, NHE1, and Hv1

Voltage-gated sodium channels can enhance invasive behavior in non-excitable cancer cells. The most developed evidence concerns a neonatal splice variant of Nav1.5/SCN5A in breast and colon cancer, where persistent Na^+^ entry couples to NHE1, extracellular acidification, invadopodial proteolysis, and metastasis [161,162,163,164]. Nav1.7/SCN9A has also been linked to cancer-cell migration and pain biology, but lung-cancer-specific evidence is less mature. The extrapolation to EGFR-mutant NSCLC is therefore Level D unless direct resistant-model data are available.

NHE1 and Hv1 illustrate why the channelome should include transporters and proton conductances. Cancer cells maintain a relatively alkaline cytosol while acidifying the extracellular compartment; this pH inversion promotes protease activity, matrix degradation, survival, and drug resistance [165,166]. Hv1 can support proton extrusion and redox balance in selected cancer models [167]. Because lysosomal acidity and autophagic flux can influence receptor degradation and persister survival, pH-regulatory systems are plausible combination targets. Yet systemic pH transport is physiologically essential, and tumor-selective therapeutic windows remain a major obstacle. For this group—Nav1.5, Nav1.7, NHE1, and Hv1—the evidence required before further investigation in EGFR-mutant NSCLC can be justified is specific and currently absent: demonstration that the transporter or channel is expressed and functionally active in osimertinib-resistant EGFR-mutant lung models rather than inferred from breast or colon data; genetic loss-of-function in those models with rescue; evidence that the resistance phenotype, not merely invasion or migration, depends on it; and a tolerated exposure in vivo at which the relevant current or transport activity is measurably suppressed. Until those four are available, the extrapolation remains Level D, and these targets should not enter combination planning.

Table 4 ranks ion-channel and transporter targets by biophysical function, representative modulators, evidence tier, and translational limitation.

## 8. Multi-Omics and Functional Diagnostics

### 8.1. What TCGA Can and Cannot Establish

TCGA-LUAD and other cross-sectional datasets are useful for defining baseline co-expression, copy-number alterations, lineage programs, and survival associations [168,169]. They can identify whether RTK bypass, apoptotic, metabolic, trafficking, epigenetic, or channel/transporter transcripts cluster with EGFR signaling, proliferation, EMT, hypoxia, or immune states. They cannot establish acquired osimertinib resistance because most samples predate contemporary targeted therapy and are not longitudinally paired. Any target-survival association may therefore reflect stage, proliferation, tumor purity, stromal composition, or lineage rather than treatment-specific dependency.

Analyses should therefore adjust for tumor purity, copy number, histology, stage, smoking, TP53/RB1 status, EGFR mutation subtype, and proliferation signatures. Multiple-testing correction is essential when evaluating hundreds of channel and transporter genes. External validation should use an independent cohort with treatment annotation. The most informative dataset would contain baseline tissue, serial plasma, and progression tissue from patients receiving first-line osimertinib or an intensified regimen.

### 8.2. Liquid Biopsy and Serial Clonal Measurement

ctDNA offers broad spatial sampling and repeated measurement. It can detect emerging C797S, RAS-pathway mutations, and fusions, quantify molecular response, and identify multiple concurrent clones [84,85,86,87,88,89,170,171]. However, copy-number calling is purity-dependent, and transformed histology is invisible. A rational workflow combines plasma first with tissue whenever plasma is negative or when MET amplification, SCLC transformation, or a targetable protein state is suspected.

Biomarkers for non-genomic targets are more difficult than plasma–DNA genotyping. EGFR or TROP2 abundance does not fully predict ADC response, and RNA or protein expression of AXL, TEAD programs, ion channels, or transporters does not prove functional dependency. Potential approaches include serial tissue proteomics, extracellular-vesicle RNA, circulating tumor cells, functional electrophysiology, and short-term organoid perturbation. Each assay requires analytical validation and must distinguish target abundance from target engagement and drug response. The standard a functional assay has to meet is the operational definition given at the head of Section 7: a change in viability after inhibitor treatment is not sufficient evidence of dependency on its own, and must be accompanied by genetic loss-of-function, rescue by re-expression of the wild-type protein, and reproduction of the phenotype by a structurally unrelated agent at an exposure compatible with measured target engagement. Section 9 sets out the staged development framework in which these criteria must be satisfied.

### 8.3. Single-Cell, Spatial, Functional, and Computational Integration

Single-cell RNA sequencing has revealed therapy-induced evolution and coexistence of multiple resistant states in patient-derived lung cancer samples [45]. Complementary microenvironmental and spatial analyses add the context of whether a target-high state is localized near fibroblasts, hypoxic niches, vessels, immune aggregates, or invasive edges [90,91]. Yet transcript abundance remains an imperfect surrogate for membrane current or pathway dependency. Integration with surface proteomics, phosphoproteomics, calcium imaging, electrophysiology where relevant, and drug perturbation is needed to connect molecular state to pharmacological function.

Computational models can integrate histology, radiology, clinical variables, ctDNA trajectories, multi-omic features, and perturbational-response signatures [172,173,174,175], but their value depends on prospective calibration and treatment-linked endpoints. Static pretreatment data are unlikely to predict every evolutionary path. Models should therefore be updated longitudinally and judged by decision utility: whether they improve selection among continued TKI with local therapy, MET-directed combinations, chemotherapy, bispecific antibodies, ADCs, or a clinical trial. Prediction without an externally validated assay, prespecified intervention, and prospective clinical endpoint should remain hypothesis-generating.

## 9. Translational Pharmacology Framework for Mechanism-Matched Combinations

The most defensible development path is not to add a second drug empirically to osimertinib. It is to define a reproducible, druggable dependency; demonstrate target engagement at tolerated exposure; and test the combination in a biomarker-enriched population against a contemporary standard. The framework applies to RTK bypass, downstream signaling, lineage-associated targets, apoptosis, autophagy, and ion-channel or transporter programs.

- Discovery: analyze paired baseline and progression tissue from first-line osimertinib, chemotherapy-intensified, and EGFR-MET bispecific-treated cohorts using DNA, RNA, spatial protein, phosphoproteomics, and lineage assays.
- Causality: reproduce the patient-associated state in isogenic systems, organoids, and PDXs; combine genetic loss, rescue, orthogonal chemotypes, and direct target-engagement assays.
- Pharmacology: quantify unbound exposure, time above effective concentration, CNS penetration, pathway suppression, combination matrices, schedule dependence, and organ-specific safety margins.
- Biomarker assay: develop a clinically deployable genomic, protein, RNA, or functional assay with a prespecified threshold, reproducibility, spatial robustness, and actionable turnaround time.
- Early clinical trial: use a mechanism-stratified phase I or window design with paired biopsies, pharmacokinetic-pharmacodynamic modeling, serial ctDNA, and proof of target modulation rather than objective response alone.
- Randomized validation: compare the mechanism-matched combination with the relevant current standard and include CNS PFS, OS, patient-reported outcomes, molecular clearance, and resistance at progression.

Randomized validation must compare the proposed combination with contemporary standards and incorporate target-specific safety. EGFR-MET regimens introduce dermatologic, thrombotic, and infusion-related toxicity; ADCs add payload-dependent ILD, cytopenias, stomatitis, ocular effects, and cumulative marrow exposure. SHP2, MEK, PI3K, autophagy, apoptosis, and ion-channel combinations require explicit free-exposure and schedule analyses. hERG pore blockade is generally unacceptable without tumor-selective delivery or a non-conducting target interface; SOCE and KCa3.1 modulation may alter immune function; and broad pH or sodium-channel inhibition risks normal-tissue toxicity. Figure 5 converts this evidence hierarchy into a practical progression-workflow in which clinical decisions are separated from research-only functional diagnostics.

Table 5 defines the author-proposed minimum evidence required before any compensatory target is treated as clinically actionable.

## 10. Discussion

The treatment history of EGFR-mutant NSCLC is a sequence of increasingly complete pharmacological attempts to suppress oncogene addiction. First-generation TKIs established genotype-selected therapy; second-generation inhibitors broadened ErbB blockade; osimertinib solved much of the T790M and CNS problem; chemotherapy combinations and EGFR-MET bispecific therapy now deepen first-line suppression; and ADCs deliver cytotoxic payloads across heterogeneous resistance states. Each advance nevertheless leaves residual populations shaped by the new exposure environment, so the architecture of resistance becomes more diverse rather than simply more potent.

The strongest current evidence supports a target hierarchy. On-target C797S or compound EGFR, mutations justify mutant-selective or allosteric development when allelic context and CNS exposure are favorable. MET amplification is the best-established bypass dependency and supports EGFR-MET combinations. Histologic transformation requires lineage-directed treatment. When no single driver dominates, chemotherapy, amivantamab-based regimens, and ADCs offer mechanism-spanning activity. These categories should be distinguished from lower-maturity targets whose evidence is confined to conventional cell lines or cross-tumor inference.

Emerging targets such as AXL, SHP2, MEK, PI3K-mTOR, YAP/TEAD, autophagy, lysosomal programs, apoptotic regulators, ion channels, and pH transporters are pharmacologically plausible but not interchangeable. AXL and YAP-linked states may identify reversible persistence; SHP2 can suppress convergent RTK-RAS signaling but may trigger adaptive feedback; autophagy and BCL-2-family dependence vary with treatment history; and channel or transporter expression may reflect lineage rather than causal dependency. For each class, the five pharmacological criteria defined in Section 2 and operationalized stage by stage in Table 5 are more informative than nominal inhibitor sensitivity.

ANO1 illustrates both the opportunity and the risk of a candidate biophysical target. Direct ANO1-EGFR interaction and downstream signaling effects have been demonstrated in selected epithelial cancer models [131,132], but these are head-and-neck and breast models; they establish mechanistic plausibility (Level D for the EGFR-mutant lung setting) and do not establish a causal role in acquired osimertinib resistance, for which longitudinal evidence in EGFR-mutant NSCLC remains insufficient. A further unresolved point is whether the relevant phenotype depends on chloride conductance through the pore, on a non-conducting scaffolding interaction with EGFR, or on both; the two possibilities imply different therapeutic modalities, since a pore blocker and a protein–protein interaction disruptor would not be interchangeable. Discriminating between them requires conductance-dead point mutants that retain the interaction, and interaction-deficient mutants that retain conductance, tested against the same phenotype. It is appropriate to investigate ANO1-dependent conductance, receptor organization, and trafficking; it is not appropriate to describe ANO1 as a validated or even an established dependency in this setting. The same evidentiary discipline is applied below to KCa3.1-SOCE coupling, hERG, LRRC8/VRAC, TRP channels, Nav-NHE1, and proton conductances.

Several apparently conflicting findings are explained by pharmacological and sampling design. C797S frequency differs between first- and later-line osimertinib because the latter selects a T790M-enriched population. MET amplification differs between plasma and tissue because copy-number calling depends on tumor fraction and threshold. Transformation rates are higher in cohorts that mandate tissue biopsy. ADC efficacy is not reducible to antigen immunohistochemistry because internalization, linker cleavage, payload sensitivity, efflux, and DNA-damage response contribute. Channel studies disagree because cell-line backgrounds, culture conditions, free concentrations, inhibitor selectivity, and functional assays differ.

A practical route forward is a mechanism-informed functional platform. A progression biopsy can be allocated to histology, DNA/RNA sequencing, spatial proteomics, and short-term organoid testing. A focused perturbation panel can test EGFR, MET, AXL, SHP2, MEK, PI3K-mTOR, autophagy, apoptosis, and selected biophysical targets using clinically achievable exposures and schedule-aware combinations. Serial ctDNA and paired pharmacodynamic tissue can then determine whether the regimen suppresses the relevant clone or merely produces transient cytostasis.

The principal obstacles are biopsy feasibility, spatial heterogeneity, model selection, poor tool-compound selectivity, and competition with rapidly improving standards. A new combination must show more than Bliss or Loewe synergy in vitro; the go/no-go criteria it has to satisfy are those set out stage by stage in Table 5, and the last of them—a clinically meaningful advantage over chemotherapy, EGFR-MET therapy, or ADCs—is the hardest to meet. These requirements are especially stringent for systemic ion-channel inhibition, where the therapeutic target may also be a cardiac, neurologic, immune, or secretory liability.

Table 6 consolidates major discordances and provides conservative wording that can reduce overinterpretation during peer review.

Table 7 consolidates, in a single view, the information that is otherwise distributed across Section 4, Section 5, Section 7 and Section 8 and across Table 2, Table 3 and Table 4: the treatment context in which each resistance state arises, the sampling compartment and assay that can identify it, the mechanism-matched action available, and the independently appraised evidence for the putative mechanism versus the treatment strategy. It is intended as the practical key to the review. Evidence levels are those defined in Section 2 and are reported separately for clinical and mechanistic maturity.

## 11. Conclusions

EGFR-mutant NSCLC has evolved into a pharmacologically diverse treatment system that includes mutation-selective TKIs, first-line intensification, bispecific antibodies, ADCs, and lineage-directed therapy. The remaining barrier is not simply insufficient kinase potency; it is the coexistence of on-target mutations, receptor bypass, convergent signaling, phenotypic plasticity, and reversible survival states.

The clinically strongest compensatory targets are currently EGFR itself, MET, lineage-defined transformation, and validated ADC delivery antigens. AXL, SHP2, TEAD, autophagy, and apoptosis regulators are less mature. Ion channels and transporters are candidate targets rather than established dependencies: none currently meets the operational definition given in Section 7, none has Level A or Level B clinical evidence in this setting, and none is clinically actionable today. ANO1, LRRC8/VRAC, KCa3.1-SOCE, hERG, Nav-NHE1, and related systems should be advanced only through exposure-linked, target-engagement-based validation in paired human and patient-derived models.

Durable control will require integration of three dimensions: genomic mechanism, lineage or epigenetic state, and functional pharmacological dependency. Prospective studies combining paired biopsy, serial ctDNA, pharmacokinetic–pharmacodynamic modeling, organoid perturbation, and biomarker-defined treatment can determine whether a combination truly eliminates a resistant clone. This approach converts the channelome and other emerging compensatory pathways from descriptive associations into testable, falsifiable drug-development programs.

## Figures and Tables

**Figure 1:**
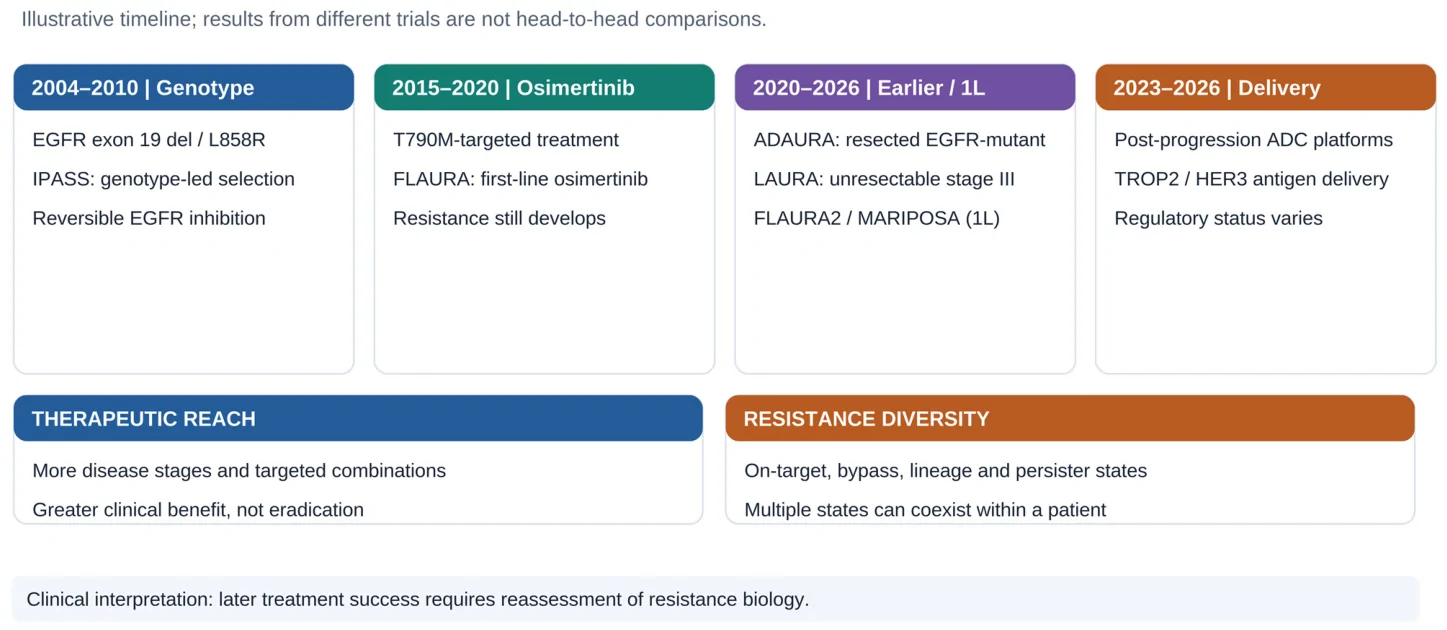
Evolution of EGFR-directed treatment and residual resistance. Four periods illustrate the shift from genotype-directed EGFR inhibition to osimertinib, earlier-stage and first-line intensification, and subsequent antibody-drug conjugate platforms. FLAURA2 and MARIPOSA are included as first-line intensification trials; their placement does not imply equivalent endpoints or approval status. The lower panels contrast widening therapeutic reach with heterogeneous residual resistance. The timeline is schematic, and cross-trial comparisons should not be inferred. Terms: EGFR, epidermal growth factor receptor; 1L, first-line; exon 19 del, exon 19 deletion; ADC, antibody–drug conjugate; T790M, EGFR gatekeeper substitution. IPASS denotes genotype-selected first-line treatment; FLAURA, first-line osimertinib; ADAURA, adjuvant treatment after surgery; LAURA, post-chemoradiotherapy consolidation; FLAURA2 and MARIPOSA, first-line intensification. The illustrated therapeutic reach and resistance diversity are conceptual, not numerical efficacy metrics. Original figure. The scientific content, layout, and all data labels are the author’s; the artwork was redrawn as vector graphics for this revision to correct labelling errors and to meet the journal’s resolution requirement. No figure was produced by a text-to-image generative model; the tools used are disclosed in the Acknowledgments.

**Figure 2:**
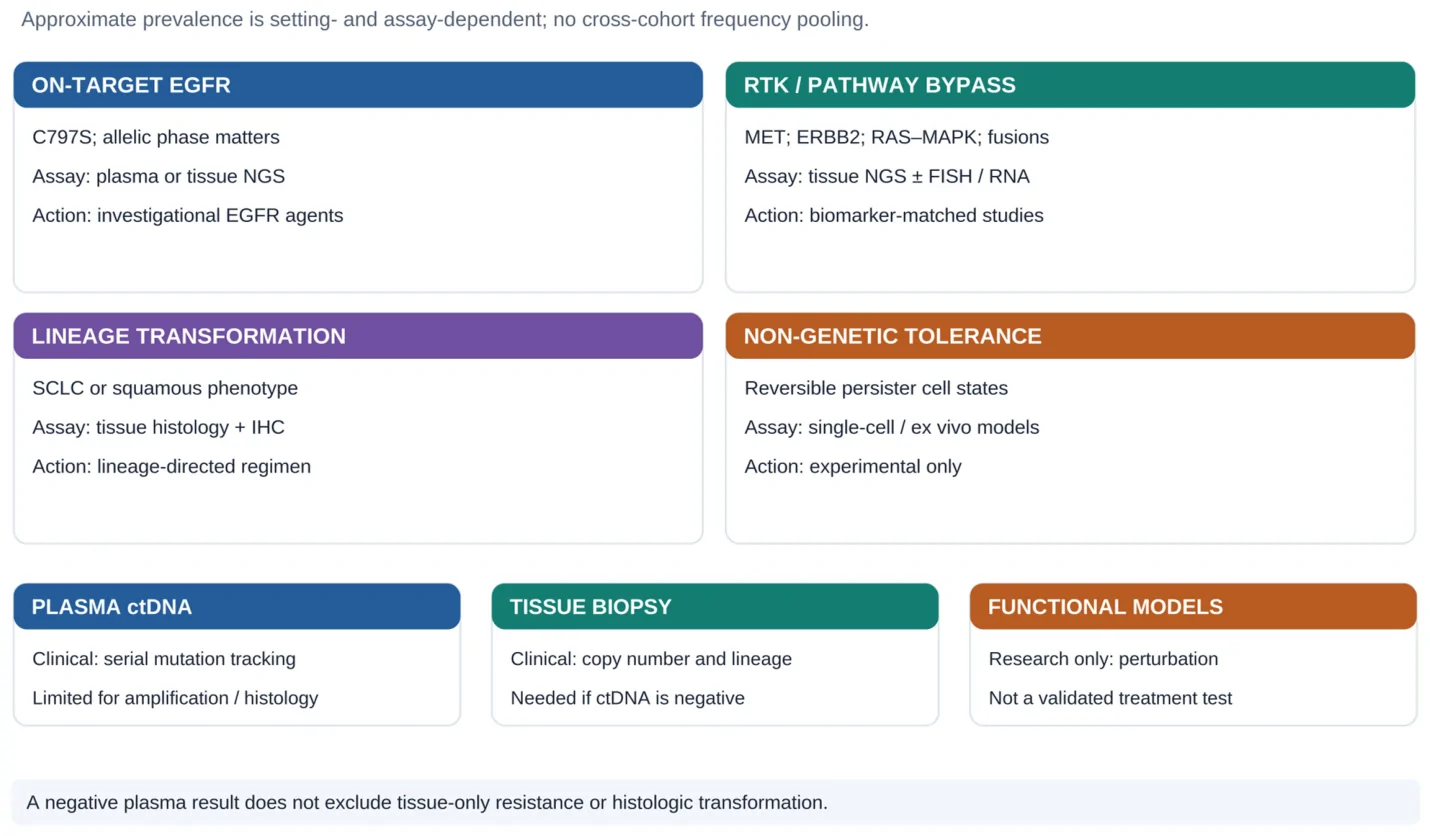
Assay-aware atlas of resistance after osimertinib. Four mechanism classes are distinguished: on-target EGFR changes, receptor/pathway bypass, lineage transformation, and non-genetic tolerance. The figure identifies appropriate assays and illustrative treatment logic without assigning pooled resistance frequencies across incompatible cohorts. The lower row distinguishes routine plasma and tissue sampling from research-only functional models. Plasma testing does not replace tissue histology when transformation is suspected. Assay labels: NGS, next-generation sequencing; FISH, fluorescence in situ hybridization; RNA, RNA-based fusion testing; IHC, immunohistochemistry; ctDNA, circulating tumor DNA; SCLC, small-cell lung cancer; RTK, receptor tyrosine kinase; RAS–MAPK, RAS/mitogen-activated protein kinase pathway. Molecular tests can detect substitutions or amplifications, whereas tissue morphology and immunophenotyping are needed to diagnose lineage transformation. Single-cell and ex vivo assays represent research, not standard clinical diagnostic pathways. Original figure. The scientific content, layout, and all data labels are the author’s; the artwork was redrawn as vector graphics for this revision to correct labelling errors and to meet the journal’s resolution requirement. No figure was produced by a text-to-image generative model; the tools used are disclosed in the Acknowledgments.

**Figure 3:**
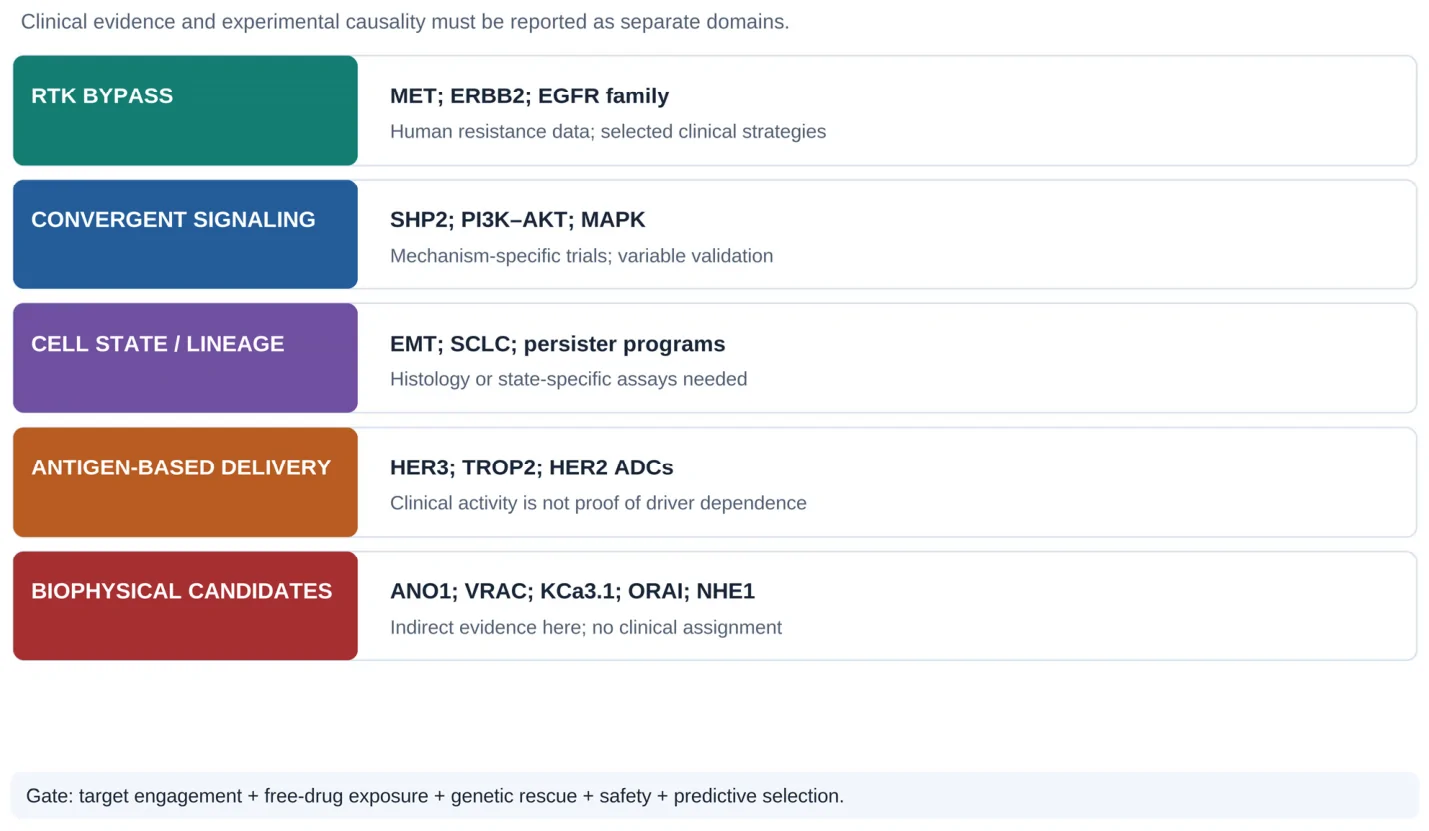
Compensatory pharmacology after EGFR-directed therapeutic pressure. Receptor-level bypass, convergent signaling, lineage and persister programs, antigen-directed delivery, and biophysical candidate targets are presented according to their distinct clinical and experimental evidence. ADC efficacy does not establish target antigen dependence as a resistance driver. Biophysical modifiers remain research-layer candidates. The go/no-go statement summarizes target engagement, exposure, genetic rescue, safety, and biomarker selection as development requirements developed in Section 9. RTK refers to receptor tyrosine kinases; MET and ERBB2 represent receptor-level bypass, SHP2/PI3K–AKT/MAPK are convergent signaling programs, EMT is epithelial–mesenchymal transition, SCLC is small-cell lung cancer, ADC is an antibody–drug conjugate, and ANO1/VRAC/KCa3.1/ORAI/NHE1 are biophysical candidate classes. Level A means prospective/randomized clinical validation in EGFR-mutant NSCLC; B means non-randomized human evidence; C means causal experimental evidence in relevant EGFR-mutant NSCLC models; D means indirect evidence from other tumor types or normal physiology. Unlike the earlier artwork, the revised schematic intentionally contains no mixed labels such as A/B, B–C, or C–D because such labels ambiguously combine clinical and mechanistic evidence domains. When both domains are reported in the text and tables, they are explicitly named separately, not merged into one score. Original figure. The scientific content, layout, and all data labels are the author’s; the artwork was redrawn as vector graphics for this revision to correct labelling errors and to meet the journal’s resolution requirement. No figure was produced by a text-to-image generative model; the tools used are disclosed in the Acknowledgments.

**Figure 4:**
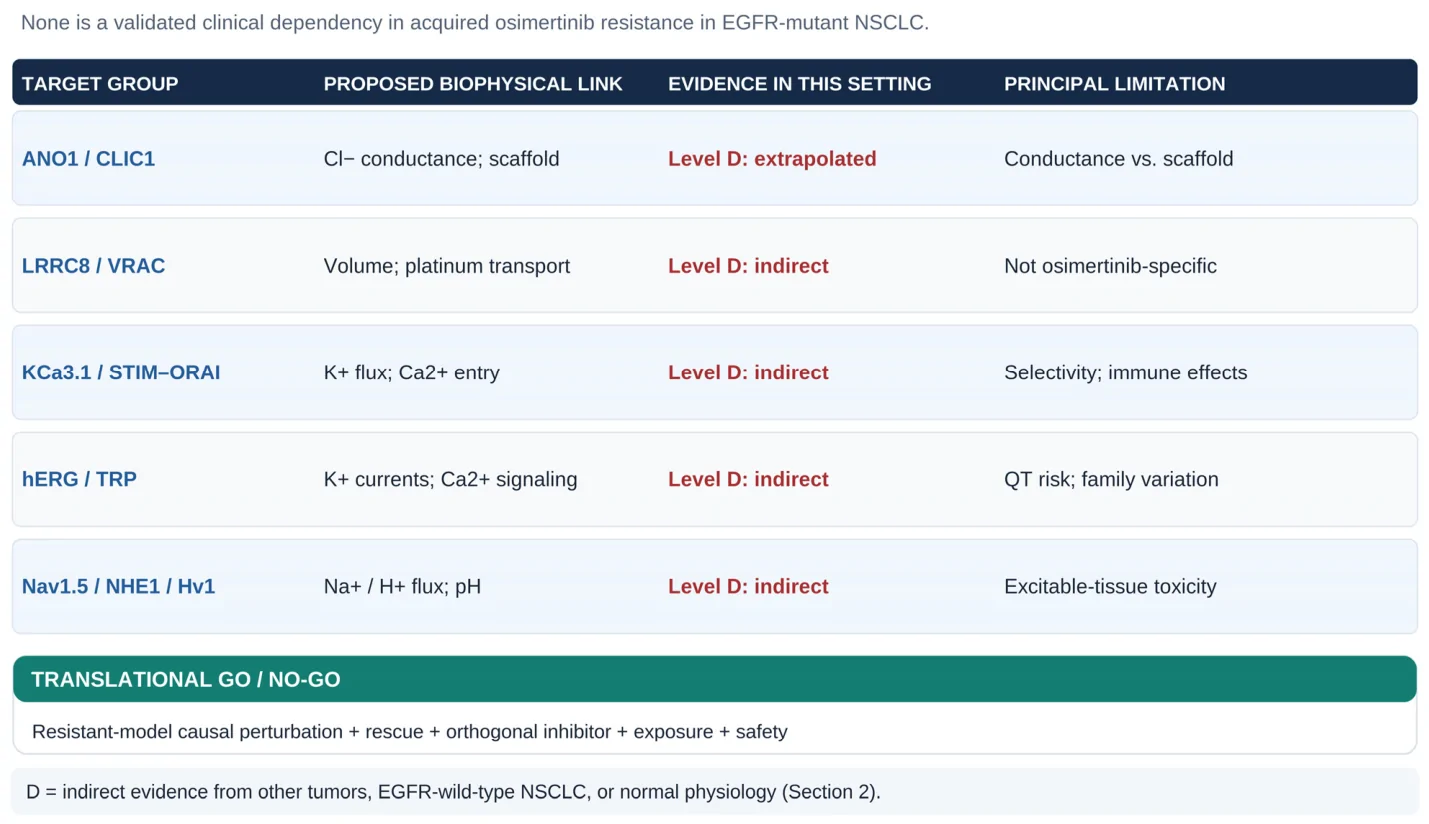
Biophysical candidate-target groups and translational requirements in EGFR-mutant NSCLC. ANO1/CLIC1, LRRC8/VRAC, KCa3.1/STIM–ORAI, hERG/TRP, and Nav/NHE1/Hv1 are grouped by proposed function rather than by established acquired resistance to osimertinib. The codes refer to this disease-specific question: Level D is indirect evidence from other tumors, EGFR-wild-type NSCLC, or normal physiology, and Level A/B clinical validation or Level C experimental causal evidence of acquired osimertinib resistance has not been demonstrated for the groups shown. No group shown qualifies as a clinical treatment dependency. ANO1, anoctamin 1 (TMEM16A); CLIC1, chloride intracellular channel 1; LRRC8/VRAC, leucine-rich repeat-containing protein 8/volume-regulated anion channel; KCa3.1, calcium-activated potassium channel 3.1; STIM–ORAI, store-operated calcium entry machinery; hERG, human ether-à-go-go-related gene potassium channel; TRP, transient receptor potential channels; Nav1.5, voltage-gated sodium-channel alpha subunit; NHE1, sodium/hydrogen exchanger 1; Hv1, voltage-gated proton channel. Level A is prospective/randomized clinical validation, B is observational human evidence, C is EGFR-mutant NSCLC-specific causal experimental evidence, and D is indirect evidence. All depicted target groupings are classified as Level D for acquired osimertinib resistance; none is yet clinically actionable. QT denotes cardiac repolarization interval. Colors are used only for visual grouping and do not encode evidence strength or quantitative information. Original figure. The scientific content, layout, and all data labels are the author’s; the artwork was redrawn as vector graphics for this revision to correct labelling errors and to meet the journal’s resolution requirement. No figure was produced by a text-to-image generative model; the tools used are disclosed in the Acknowledgments.

**Figure 5:**
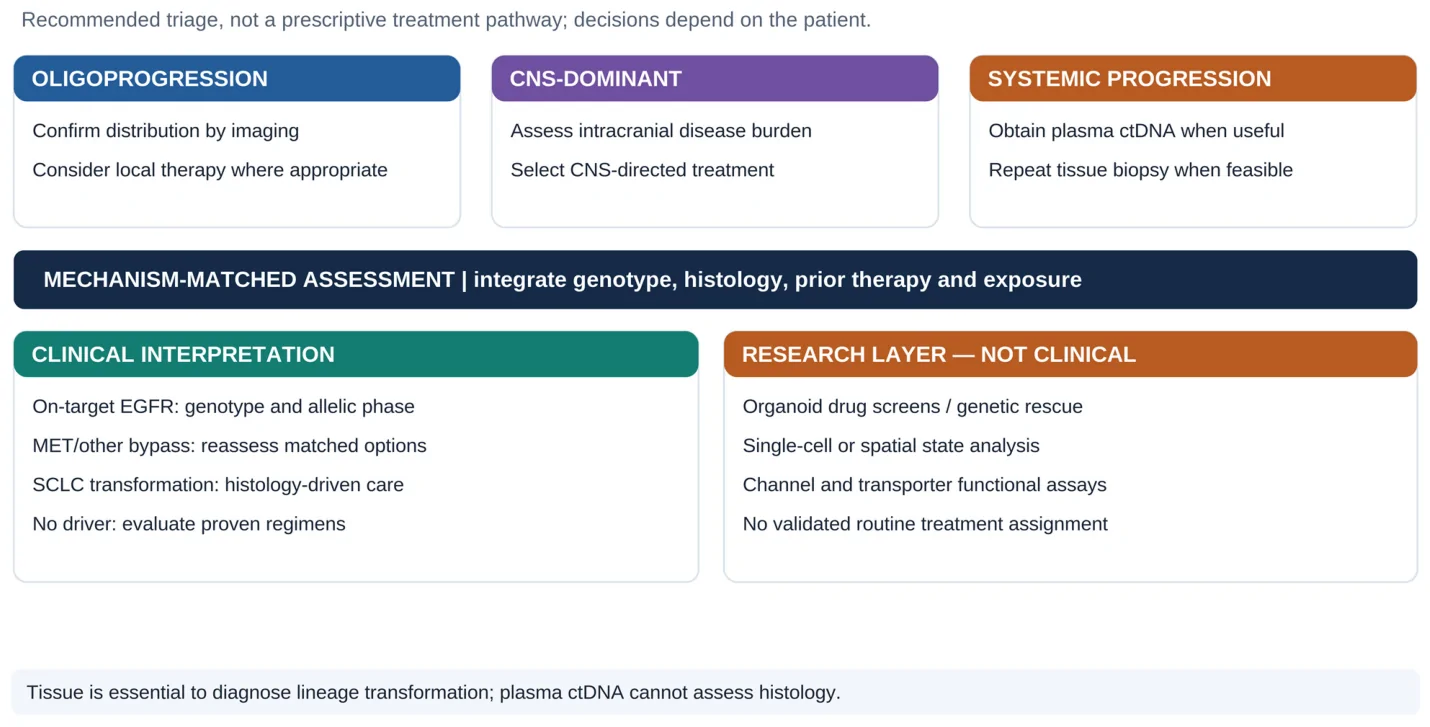
Mechanism-informed reassessment after progression on EGFR-directed therapy. Oligoprogression, CNS-dominant progression, and systemic progression prompt different clinical assessments. Genomic profiling and repeat tissue biopsy inform mechanism- and histology-matched treatment selection. Organoid perturbation, single-cell, or spatial analysis, and channel/transporter functional studies are clearly separated as research-only approaches; they are not validated routine treatment-selection tests. CNS, central nervous system; ctDNA, circulating tumor DNA; NGS, next-generation sequencing; SBRT, stereotactic body radiotherapy; SRS, stereotactic radiosurgery; ADC, antibody–drug conjugate; PK/PD, pharmacokinetics/pharmacodynamics; SCLC, small-cell lung cancer. Clinical arrows indicate reassessment of progression pattern, plasma/tissue profiling, and selection of treatment supported by the verified disease and mechanism. Functional organoid, spatial, and channel assays are explicitly research-only and should not be interpreted as validated treatment-selection tests. Original figure. The scientific content, layout, and all data labels are the author’s; the artwork was redrawn as vector graphics for this revision to correct labelling errors and to meet the journal’s resolution requirement. No figure was produced by a text-to-image generative model; the tools used are disclosed in the Acknowledgments.

**Table 1:** Landmark trials that changed the EGFR-mutant NSCLC treatment pathway. Values are quoted from the primary publication indicated and, where overall survival was reported in a later analysis, from that report; the assessment method (investigator-assessed or blinded independent central review) is stated because medians are not interchangeable between them. Entries are descriptive context and are not direct cross-trial comparisons. Abbreviations: AE, adverse event; BICR, blinded independent central review; CI, confidence interval; DFS, disease-free survival; EGFR, epidermal growth factor receptor; Ex19del, exon 19 deletion; HR, hazard ratio; N, total trial enrollment; NSCLC, non-small-cell lung cancer; ORR, objective response rate; OS, overall survival; PFS, progression-free survival; TKI, tyrosine kinase inhibitor.

Study	Population	Comparison	*N*	Key Quantitative Result	Interpretation	Ref.
IPASS	Untreated clinically selected pulmonary adenocarcinoma; EGFR-mutant subgroup	Gefitinib vs. carboplatin-paclitaxel	1217 overall	Mutant subgroup PFS HR 0.48 (95% CI 0.36–0.64; investigator-assessed); ORR 71.2% vs. 47.3%	Established genotype as the predictive variable	[9]
AURA3	T790M-positive after EGFR TKI	Osimertinib vs. platinum-pemetrexed	419	PFS 10.1 vs. 4.4 mo (investigator-assessed); HR 0.30 (95% CI 0.23–0.41); ORR 71% vs. 31%	Third-generation TKI replaces chemotherapy in T790M-positive disease	[19]
FLAURA	Untreated Ex19del/L858R advanced NSCLC	Osimertinib vs. gefitinib/erlotinib	556	PFS 18.9 vs. 10.2 mo (investigator-assessed); HR 0.46 (95% CI 0.37–0.57); OS 38.6 vs. 31.8 mo; HR 0.80 (95.05% CI 0.64–1.00)	First-line standard; strong CNS control	[20,21]
ADAURA	Completely resected stage IB-IIIA EGFR-mutant NSCLC	Osimertinib vs. placebo	682	DFS HR 0.17 (stage II–IIIA, primary; 99.06% CI 0.11–0.26) and 0.20 (stage IB–IIIA; 99.12% CI 0.14–0.30), investigator-assessed; updated final DFS HR 0.23 (stage II–IIIA; 95% CI 0.18–0.30) and 0.27 (overall; 95% CI 0.21–0.34), also investigator-assessed; 5-y OS 88% vs. 78% (stage IB–IIIA); death HR 0.49 (95.03% CI 0.34–0.70)	Demonstrated curative-intent benefit; optimal duration and post-recurrence sequencing remain open	[22,23,83]
FLAURA2	Untreated advanced Ex19del/L858R NSCLC	Osimertinib + platinum-pemetrexed vs. osimertinib	557	PFS 25.5 vs. 16.7 mo (investigator-assessed; HR 0.62, 95% CI 0.49–0.79); OS 47.5 vs. 37.6 mo (HR 0.77, 95% CI 0.61–0.96, *p* = 0.02); 3-y OS 63% vs. 51%	Broad intensification; higher grade 3–4 hematologic toxicity	[24,70]
LAURA	Unresectable stage III after chemoradiotherapy without progression	Osimertinib vs. placebo	216	PFS 39.1 vs. 5.6 mo (BICR); HR 0.16 (95% CI 0.10–0.24); interim OS not significant	Defines consolidation standard; long-term OS and resistance biology continue to mature	[25]
MARIPOSA	Untreated advanced Ex19del/L858R NSCLC	Amivantamab-lazertinib vs. osimertinib	1074	PFS 23.7 vs. 16.6 mo (BICR; HR 0.70, 95% CI 0.58–0.85); OS NR vs. 36.7 mo (HR 0.75, 95% CI 0.61–0.92, *p* = 0.005); 3-y OS 60% vs. 51%; grade 3+ AEs 80% vs. 52% (OS analysis)	First-line dual EGFR-MET strategy; toxicity and treatment burden are important	[26,71]
PAPILLON	Untreated advanced EGFR exon 20 insertion NSCLC	Amivantamab + carboplatin-pemetrexed vs. chemotherapy	308	PFS 11.4 vs. 6.7 mo (BICR); HR 0.40 (95% CI 0.30–0.53)	Establishes combination standard for exon 20 insertion disease	[77]

**Table 2:** Resistance mechanisms after osimertinib and sources of discordant frequency estimates. Frequencies are not directly comparable across rows: they derive from different treatment lines, biopsy-selected or plasma-screened populations, and assay platforms, and the sampling compartment that can detect each mechanism is stated for that reason.

Mechanism	Reported Frequency	Best Detection Method	Functional Consequence	Critical Caveat	Ref.
EGFR C797S	Typically 5–15%; higher after later-line than first-line osimertinib	Plasma or tissue NGS	Loss of covalent Cys797 binding	Allelic phase with T790M changes combination sensitivity	[33,34,37,38,39]
Other EGFR mutations/amplification	Usually each <5%; aggregate clinically relevant	NGS; copy-number analysis	Conformational change or increased receptor dosage	Mutation-specific pharmacology; not a single class	[39,82,93,94,95]
MET amplification	Approximately 10–20%	Tissue NGS/FISH; plasma less sensitive	ERBB3-PI3K-AKT bypass; HGF may activate without amplification	Thresholds differ across trials and assays	[27,28,29,36,37,38,39]
ERBB2 amplification/PI3K-RAS-MAPK	Usually low single digits for each event	Tissue/plasma NGS	Parallel survival signaling	Often subclonal; combination toxicity and pathway redundancy	[30,36,37,38,39]
Oncogenic fusions	Rare, collectively several percent	RNA or DNA NGS	Alternative kinase dependency	RNA assays improve detection; evidence often from case series	[36,37,38,39]
SCLC transformation	Approximately 5–15% in tissue-biopsied cohorts	Tissue morphology + neuroendocrine markers	Lineage switch; frequent RB1/TP53 loss	Invisible to plasma-only testing; retains founding EGFR mutation	[51,52,53,54,55,56]
Squamous transformation/EMT	Variable; enriched in tissue-based cohorts	Histology; RNA/protein state markers	Lineage plasticity and altered apoptotic dependence	Marker definitions and sampling time vary	[38,49,55]
Drug-tolerant persistence	Not captured as a fixed clinical frequency	Serial single-cell, ex vivo functional assays	Reversible chromatin, AXL, YAP, autophagy, metabolism	State is dynamic; most evidence is preclinical	[40,41,42,43,44,45,46,47,48,49,50]

**Table 3:** Selected pharmacological strategies after osimertinib progression. The study design is given with each study name because randomized, single-arm, early-phase, and pooled regulatory analyses are not comparable; response rates from non-randomized studies must not be read against randomized outcomes.

Study/Program	Regimen	Population/Biomarker	Quantitative Efficacy Signal	Limitations	Ref.
TATTON (phase Ib, single-arm expansion cohorts)	Osimertinib + savolitinib	EGFR-mutant, MET-driven resistance after prior EGFR TKI	ORR 33% (Part B1, prior third-generation TKI) to 67% (Part B3); 62% in Part D; activity depended on prior third-generation TKI exposure and on how MET positivity was defined	Non-randomized phase Ib cohorts; heterogeneous MET assays; not comparable with randomized data	[100,101]
INSIGHT 2 (phase II, single-arm)	Tepotinib + osimertinib	MET-amplified after first-line osimertinib	ORR 50.0% (central FISH^+^, 95% CI 39.7–60.3); median PFS 5.6 months (95% CI 4.2–8.1)	Phase II; biomarker threshold and tissue/plasma concordance remain important	[102]
MARIPOSA-2 (randomized phase III)	Amivantamab + chemotherapy with/without lazertinib	After progression on osimertinib	BICR PFS 6.3 mo (amivantamab-chemotherapy; HR 0.48, 95% CI 0.36–0.64) and 8.3 mo (amivantamab-lazertinib-chemotherapy; HR 0.44, 95% CI 0.35–0.56) vs. 4.2 mo (chemotherapy); ORR 64%, 63%, and 36%, respectively	Higher hematologic toxicity; regimen and prophylaxis evolved during study	[107]
HER3-DXd (phase I dose expansion, 5.6 mg/kg, *n* = 57)	Patritumab deruxtecan	EGFR TKI-resistant EGFR-mutant NSCLC	Confirmed ORR 39% (95% CI 26.0–52.4, BICR); median PFS 8.2 mo	No validated HER3-expression threshold; ILD and cytopenias	[109]
HERTHENA-Lung01 (phase II, single-arm, *n* = 225)	Patritumab deruxtecan	After EGFR TKI and platinum chemotherapy	Confirmed ORR 29.8% (95% CI 23.9–36.2, BICR); PFS 5.5 mo; OS 11.9 mo	Single-arm; US application withdrawn 29 May 2025, following HERTHENA-Lung02 results; OS was immature in cited ASCO abstract	[110,112,113]
TROPION-Lung05 (phase II, single-arm) and TROPION-Lung01 (randomized phase III); regulatory pooled EGFR cohort	Datopotamab deruxtecan	Actionable-genomic-alteration NSCLC after targeted therapy and platinum	Regulatory pooled EGFR-mutant cohort *n* = 114: ORR 45% (95% CI 35–54), DOR 6.5 mo (95% CI 4.2–8.4); accelerated approval 23 June 2025. A separately published pool (*n* = 117) reported ORR 43% and DOR 7.0 mo and is not interchangeable	Stomatitis, ocular toxicity, ILD; confirmatory benefit required	[114,115,116,117]
Fourth-generation TKI trials (phase I/II, early)	Allosteric or mutant-selective agents	C797S/compound EGFR mutations	Molecular responses in selected early-phase cases	Small numbers; CNS exposure, allelic context, and development status vary	[93,94,95,96,97,98,99]

**Table 4:** Evidence-ranked ion-channel and transporter candidate targets relevant to EGFR-resistant NSCLC. Evidence levels follow the definitions in Section 2 and are reported separately for clinical and mechanistic maturity. The Level D assignments concern evidence for acquired resistance to osimertinib in EGFR-mutant NSCLC; functional data from other cancer settings do not raise that grade. No entry in this table currently meets the operational definition of a dependency given in Section 7; all are candidate targets or associations.

Target	Biophysical Function	Key Experimental Evidence	Representative Modulator	Evidence Level (Clinical/Mechanistic)	Translational Limitation	Ref.
ANO1/TMEM16A	Ca^2+^-activated Cl^−^ channel; possible scaffold	Direct EGFR co-complex in head-and-neck models; EGFR/CAMK signaling in breast models; NSCLC expression/perturbation evidence less developed	Ani9 (reported potent ANO1 inhibition); T16Ainh-A01 and other tool compounds	No clinical evidence/Level D (indirect; EGFR-mutant osimertinib resistance unvalidated)	No paired clinical post-osimertinib validation; pore versus scaffold function unresolved	[125,126,127,128,129,130,131,132,133,134]
CLIC1	Metamorphic chloride-associated protein; redox/scaffold functions	Proliferation, migration, stem-like states in several tumors; limited direct EGFR-TKI data	IAA-94 and related tool compounds are poorly selective	No clinical evidence/Level D (indirect; EGFR-mutant osimertinib resistance unvalidated)	Channel identity and off-target pharmacology complicate interpretation	[135,136]
LRRC8/VRAC	Volume regulation; osmolyte and drug transport	VRAC subunits alter platinum transport and response in experimental systems; not shown to drive osimertinib resistance	DCPIB and genetic subunit manipulation	No clinical evidence/Level D (indirect; EGFR-mutant osimertinib resistance unvalidated)	Not a demonstrated osimertinib-resistance mechanism	[138,139,140]
KCa3.1/KCNN4	K^+^ efflux hyperpolarizes membrane and sustains Ca^2+^ entry	Cancer proliferation/migration across models; limited EGFR-mutant lung validation	TRAM-34; senicapoc	No clinical evidence/Level D (indirect; EGFR-mutant osimertinib resistance unvalidated)	Repurposing hypothesis; no established phase II oncology program	[62,63,64,141,142,143,144,145]
hERG/KCNH2	Voltage-gated K^+^ channel; adhesion and survival complexes	Expression and functional data in several cancers	Astemizole and many nonselective blockers	No clinical evidence/Level D (indirect; EGFR-mutant osimertinib resistance unvalidated)	QT prolongation and torsades risk; cardiac antitarget	[144,145,146,147,148]
STIM1-ORAI1/3	Store-operated Ca^2+^ entry	Store-operated calcium entry regulates proliferation and migration in tumor models; NSCLC chemotherapy links are not osimertinib-resistance causality	SOCE inhibitors and genetic depletion	No clinical evidence/Level D (indirect; EGFR-mutant osimertinib resistance unvalidated)	Selectivity and immune effects; limited longitudinal EGFR data	[149,150,151,152,153,154,155,156]
TRP channels	Polymodal Ca^2+^/Na^+^ entry; mechanosensation/redox sensing	Family-specific effects on proliferation, migration, invasion	Family-dependent agonists/antagonists	No clinical evidence/Level D (indirect; EGFR-mutant osimertinib resistance unvalidated)	Cannot generalize across TRP subfamilies; many tool compounds are promiscuous	[157,158,159,160]
Nav1.5/Nav1.7	Persistent Na^+^ current; coupling to NHE1 and invasion	Strongest evidence in breast/colon; limited EGFR-mutant lung data	Tetrodotoxin-class or persistent-current blockers	No clinical evidence/Level D (indirect; EGFR-mutant osimertinib resistance unvalidated)	Excitable-tissue toxicity and isoform selectivity	[161,162,163,164]
NHE1/Hv1	pH regulation, redox balance, extracellular acidification	Matrix degradation and survival in acidic tumors	Amiloride analogues; Hv1 blockers	No clinical evidence/Level D (indirect; EGFR-mutant osimertinib resistance unvalidated)	Systemic physiology and tumor-selective delivery	[165,166,167]

**Table 5:** Author-proposed minimum pharmacological evidence required before a compensatory target is considered clinically actionable. The stage structure and the go/no-go criteria are proposed by the author for this review; they are not taken from, and have not been validated against, any published or externally endorsed development framework, and are offered as an explicit, falsifiable standard rather than as guidance.

Stage	Material/Model	Primary Question	Go/No-Go Criterion
1. Human discovery	Paired baseline/progression tissue plus serial plasma	Identify compensatory programs enriched at resistance and linked to defined genomic or lineage states	Replication in at least two cohorts; adjust for purity, proliferation, treatment line, and assay platform
2. Functional validation	Isogenic cells, resistant clones, organoids, PDXs	Genetic loss, orthogonal inhibitors, rescue, target engagement, and schedule-aware combination matrices	Demonstrate causal dependency and exposure-compatible synergy
3. Mechanism and target engagement	Imaging, phosphoproteomics, biochemical assays, electrophysiology where relevant	Define direct target modulation, pathway consequence, and adaptive feedback	Prespecified mechanistic endpoint beyond viability alone
4. Biomarker development	Genomic, RNA, protein, spatial, or functional assay	Threshold, reproducibility, turnaround time, heterogeneity, and relation to drug response	Assay must predict dependency or pharmacodynamic response, not merely expression
5. Phase I	Biomarker-positive resistant EGFR-mutant NSCLC	Safety, pharmacokinetics, paired-biopsy pharmacodynamics, ctDNA clearance, and preliminary efficacy	Contemporary backbone with target-specific cardiac, pulmonary, immune, neurologic, and marrow monitoring
6. Randomized validation	Mechanism-defined population	PFS, CNS PFS, OS, patient-reported outcomes, resistance at progression	Compare with current bispecific/chemotherapy/ADC standards

**Table 6:** Major areas of discordance and pharmacologically balanced interpretation.

Question	Apparently Conflicting Result	Likely Explanation	Recommended Wording	Ref.
C797S as the dominant resistance lesion	Frequent in later-line osimertinib; lower after first-line therapy	Treatment-line selection for T790M and different allelic contexts	State line of therapy and allelic phase; avoid a single pooled frequency	[33,34,37,38,39]
MET amplification frequency	Plasma series often report lower rates than tissue cohorts	Copy-number sensitivity depends on tumor fraction; FISH/NGS thresholds differ	Report assay and threshold; tissue remains important	[27,28,36,37,38,39]
Histologic transformation	Rates range from low single digits to >10%	Tissue-biopsy requirement and RB1/TP53 enrichment	Do not infer absence from plasma; separate SCLC and squamous transformation	[38,51,52,53,54,55,56]
HER3/TROP2 expression as ADC biomarker	Responses occur across expression levels; high expression does not guarantee benefit	Internalization, payload sensitivity, bystander effect, assay variability	Present antigen as delivery address, not necessarily the resistance driver	[109,110,114,115]
ANO1 as an EGFR-resistance driver	Direct interaction in selected epithelial cancers; insufficient longitudinal EGFR-mutant lung evidence	Tumor type, channel versus scaffold function, tool-compound selectivity	Frame as a testable mechanism; specify the missing experiments	[128,129,130,131,132,133,134]
hERG blockade as anticancer therapy	Antiproliferative effects versus major cardiac liability	Cancer-associated complexes may differ, but pore block affects cardiac IKr	Prioritize tumor-selective delivery or non-conducting interfaces	[144,145,146,147,148]
TCGA channel correlations	Associations with survival or EGFR signatures do not prove acquired resistance	Cross-sectional pretreatment sampling and confounding by lineage/purity	Use as discovery; validate in paired treated cohorts	[168,169]

**Table 7:** Consolidated matrix of treatment setting, candidate resistance mechanism or state, sampling method, and separate evidence for the mechanism and potential intervention. Levels A–D are defined in Section 2; evidence for clinical benefit from a treatment is never treated as proof that an inferred molecular resistance mechanism is causal. Rows are not mutually exclusive; the exon 20 insertion entry is an explicitly labeled comparator for primary driver biology, not an acquired-resistance state. Across rows, differences in population, assay, and prior therapy limit direct comparisons.

Treatment Context	Resistance Mechanism	Preferred Sampling and Assay	Evidence for the Mechanism (Clinical/Mechanistic)	Mechanism-Matched Action and Evidence for the Action
Progression on first-line osimertinib	EGFR C797S (T790M-independent)	Plasma NGS first; tissue NGS if plasma-negative; allelic phasing required	Level B clinical/Level C mechanistic	Fourth-generation or allosteric EGFR inhibitor; investigational only (early-phase human data; no randomized evidence)
Progression on second-line osimertinib (after a first- or second-generation TKI, T790M-positive)	EGFR C797S in cis or trans with T790M	Plasma or tissue NGS with explicit cis/trans phasing	Level B clinical/Level C mechanistic	Phase-dependent: trans configuration may respond to a first-generation plus third-generation TKI combination; cis requires fourth-generation agents (early-phase human data; no randomized evidence)
Progression on osimertinib (any line)	MET amplification or overexpression	Tissue NGS or FISH preferred; plasma underestimates copy number	Level B clinical/Level C mechanistic	Osimertinib plus savolitinib or tepotinib (B, single-arm); amivantamab-based regimens (A, randomized but not MET-selected)
Progression on osimertinib (any line)	ERBB2 amplification, PIK3CA, BRAF or KRAS alterations, acquired fusions	Paired tissue and plasma NGS; RNA-based fusion panel	Level B clinical/Level C mechanistic	Mechanism-specific trial; frequently subclonal, so benefit is unproven (C)
Progression on osimertinib (any line)	SCLC transformation	Tissue biopsy mandatory: morphology plus neuroendocrine IHC; RB1/TP53 status	Level B clinical/Level C mechanistic	Platinum-etoposide or other SCLC-directed regimen (B)
Progression on osimertinib (any line)	Squamous transformation or EMT	Tissue histology; RNA or protein state markers; no validated threshold	Level B clinical/Level C mechanistic	No validated matched therapy; managed as driver-negative disease, for which the evidence is given in the driver-negative row below
Any EGFR-directed therapy	Drug-tolerant persistence and other non-genetic tolerance	Serial single-cell or spatial profiling, ex vivo functional assay; research use only	Research-level experimental support for persistence; no prospectively validated clinical selection biomarker in this state	Biomarker-selected combinations under investigation; no clinical standard (C)
Progression after osimertinib with no dominant driver identified	No actionable alteration on paired testing	Paired plasma and tissue NGS reported as negative	Absence of an identified driver is a diagnostic category, not a Level A-validated resistance mechanism	Randomized trials support selected post-osimertinib regimens in their enrolled populations; this does not validate driver-negative status as a predictive biomarker (treatment-context evidence, not mechanism grade)
Progression after EGFR TKI and platinum chemotherapy	ADC payload delivery via HER3 or TROP2; HER2-targeted disease is genotype- and indication-specific	Antigen IHC is not predictive; HER2 genotyping required for HER2-mutant disease	Surface-antigen expression is not validated as the causal resistance mechanism or a general predictive threshold; no unified A–D mechanism grade	HER3-DXd: randomized PFS benefit, OS immature in cited interim abstract, US BLA withdrawn; Dato-DXd: regulatory and clinical data specific to EGFR-mutant previously treated disease; HER2-directed treatment applies to distinct molecular contexts. Do not pool across antigens or regimens.
Any EGFR-directed therapy	Ion-channel and transporter programs (ANO1, CLIC1, LRRC8/VRAC, KCa3.1, hERG, STIM-ORAI, TRP, Nav-NHE1)	No validated clinical assay; electrophysiology and functional testing are research-layer only	No clinical evidence/Level D indirect for acquired osimertinib resistance; no proven causal dependency	No clinically actionable intervention; investigation only, subject to the go/no-go gate in Table 5
First-line therapy for EGFR exon 20 insertion disease (biologically distinct from classical Ex19del/L858R)	Not an acquired-resistance state: exon 20 insertions are primary drivers with intrinsically reduced sensitivity to classical EGFR TKIs	Tissue or plasma NGS at diagnosis; insertion position within the loop affects drug sensitivity	Not applicable to acquired resistance; randomized PAPILLON treatment evidence in a biologically separate driver group	Primary-driver comparator only: amivantamab plus chemotherapy (PAPILLON) in exon 20 insertion NSCLC; exclude from acquired-resistance grade comparisons.

## Data Availability

No new data were created or analyzed in this study. Data sharing is not applicable to this article.

## Abbreviations

ADC, antibody-drug conjugate; AE, adverse event; BICR, blinded independent central review; CNS, central nervous system; ctDNA, circulating tumor DNA; DFS, disease-free survival; DOR, duration of response; EGFR, epidermal growth factor receptor; EMT, epithelial-mesenchymal transition; FISH, fluorescence in situ hybridization; HR, hazard ratio; IHC, immunohistochemistry; ILD, interstitial lung disease; NGS, next-generation sequencing; NSCLC, non-small cell lung cancer; ORR, objective response rate; OS, overall survival; PDX, patient-derived xenograft; PFS, progression-free survival; SOCE, store-operated calcium entry; TKI, tyrosine kinase inhibitor; TRP, transient receptor potential; VRAC, volume-regulated anion channel.

## Appendix A. Principal Search Strings

The following Boolean strings express, in reproducible form, the four search modules described in Section 2. They were applied in PubMed/MEDLINE without a start-date limit and updated to 15 June 2026. Because this is a structured narrative review rather than a systematic review, the search was iterative: these are the principal queries, not an exhaustive log of every variant run, and the results of backward and forward citation tracking are not reproduced here. They are provided so that a reader can reproduce the core of the search and are not offered as a PRISMA-compliant search record. Records were screened on title and abstract, then on full text where the record reported original quantitative or mechanistic data relevant to a claim made in this review.

Module i—EGFR-mutant NSCLC and EGFR tyrosine kinase inhibitors: (“carcinoma, non-small-cell lung”[MeSH] OR NSCLC[tiab]) AND (EGFR[tiab] OR “ErbB receptors”[MeSH]) AND (gefitinib[tiab] OR erlotinib[tiab] OR afatinib[tiab] OR dacomitinib[tiab] OR osimertinib[tiab] OR lazertinib[tiab] OR aumolertinib[tiab] OR furmonertinib[tiab] OR “fourth-generation”[tiab])

Module ii—acquired resistance mechanisms: (EGFR[tiab] AND NSCLC[tiab]) AND (“acquired resistance”[tiab] OR “drug resistance, neoplasm”[MeSH]) AND (C797S[tiab] OR T790M[tiab] OR MET[tiab] OR ERBB2[tiab] OR HER3[tiab] OR TROP2[tiab] OR KRAS[tiab] OR BRAF[tiab] OR PIK3CA[tiab] OR fusion*[tiab] OR “epithelial-mesenchymal transition”[MeSH] OR “small cell”[tiab] OR transformation[tiab] OR “drug-tolerant”[tiab] OR persister*[tiab])

Module iii—therapeutic platforms and diagnostics: (EGFR[tiab] AND NSCLC[tiab]) AND (“bispecific antibod*”[tiab] OR amivantamab[tiab] OR “antibody-drug conjugate*”[tiab] OR deruxtecan[tiab] OR “liquid biopsy”[tiab] OR “circulating tumor DNA”[MeSH] OR “minimal residual disease”[tiab] OR organoid*[tiab] OR “xenograft model antitumor assays”[MeSH] OR “single-cell analysis”[MeSH] OR “spatial transcriptomic*”[tiab])

Module iv—ion channels and transporters: (ANO1[tiab] OR TMEM16A[tiab] OR CLIC1[tiab] OR CLCA*[tiab] OR LRRC8*[tiab] OR VRAC[tiab] OR KCNN4[tiab] OR KCa3.1[tiab] OR KCNH2[tiab] OR hERG[tiab] OR STIM1[tiab] OR ORAI*[tiab] OR “TRPC”[tiab] OR “TRPV”[tiab] OR “TRPM”[tiab] OR SCN5A[tiab] OR SCN9A[tiab] OR “Nav1.5”[tiab] OR “Nav1.7”[tiab] OR SLC9A1[tiab] OR NHE1[tiab] OR HVCN1[tiab]) AND (cancer[tiab] OR tumor[tiab] OR tumour[tiab] OR neoplasm*[MeSH]) AND (lung[tiab] OR NSCLC[tiab] OR EGFR[tiab] OR resistance[tiab] OR proliferation[tiab] OR migration[tiab] OR apoptosis[tiab])

## Appendix B. Pharmacological Agents and Their Principal Mechanisms

**Table A1:** Brief pharmacological glossary for the compounds explicitly discussed in this review. Definitions describe principal molecular mechanisms or assay uses, not equivalent efficacy, selectivity, regulatory status, or clinical validation in EGFR-mutant NSCLC. Clinical contexts and supporting primary references remain as cited in Section 3, Section 4, Section 5, Section 6 and Section 7 and Table 1, Table 2, Table 3 and Table 4.

Agent (s)	Principal Mechanism or Molecular Target	Interpretive Qualifier
Gefitinib; erlotinib	Reversible ATP-competitive inhibitors of the EGFR kinase domain.	First-generation EGFR TKIs; susceptible to T790M-mediated resistance.
Afatinib; dacomitinib	Irreversible covalent EGFR-family (ERBB) kinase inhibitors.	Second-generation TKIs; broader wild-type ERBB inhibition limits tolerability.
Osimertinib; lazertinib; furmonertinib; aumolertinib	Mutant-selective, covalent third-generation EGFR TKIs targeting Cys797; activity differs by agent and setting.	Active against common sensitizing EGFR mutations and T790M; C797 substitutions may impede binding.
Sunvozertinib; mobocertinib	Irreversible EGFR inhibitors developed for exon 20 insertion variants.	Distinct molecular and therapeutic setting from acquired resistance after classical EGFR mutations; drug regulatory status varies.
EAI045; JBJ-04-125-02	Investigational allosteric EGFR inhibitors binding outside the ATP site.	Preclinical strategies for compound EGFR mutations; not established treatments.
CH7233163; BLU-945; BBT-176	Investigational mutant-selective EGFR inhibitors, including approaches directed at C797S-containing variants.	Spectrum and development status vary; clinical activity must not be inferred from preclinical results.
Brigatinib; cetuximab	Brigatinib: ATP-site multikinase inhibitor (clinically used against ALK); cetuximab: extracellular EGFR-blocking monoclonal antibody.	Investigational combinations for selected compound EGFR mutations; not a validated standard for osimertinib resistance.
Savolitinib; tepotinib; capmatinib	Selective ATP-competitive MET tyrosine kinase inhibitors.	MET-directed intervention; eligibility thresholds differ between studies.
Crizotinib	ATP-competitive inhibitor of ALK, ROS1, and MET kinases.	MET tractability evidence is context-dependent; not equivalent to MET-selected acquired EGFR-TKI resistance.
Amivantamab	EGFR–MET bispecific antibody with receptor modulation and immune-effector functions.	Antibody therapy used in specific EGFR-mutant populations, alone, or in combinations depending on indication.
Bevacizumab; ramucirumab	Bevacizumab neutralizes VEGF-A; ramucirumab blocks VEGFR2.	Antiangiogenic intensification; different targets and combination contexts.
Patritumab deruxtecan (HER3-DXd)	HER3-targeted antibody–drug conjugate with a cleavable linker and topoisomerase-I inhibitor payload.	Antigen-directed cytotoxic delivery; HER3 expression is not proof of an oncogenic resistance dependency.
Datopotamab deruxtecan (Dato-DXd)	TROP2-targeted antibody–drug conjugate carrying a topoisomerase-I inhibitor payload.	Antigen-mediated delivery; response depends on additional payload and tumor-state factors.
Trastuzumab deruxtecan (T-DXd)	HER2-targeted antibody–drug conjugate carrying a topoisomerase-I inhibitor payload.	Evidence and indication depend on HER2 biomarker context; do not conflate with ERBB2 amplification.
Telisotuzumab vedotin	c-MET-targeted antibody–drug conjugate with a microtubule-disrupting monomethyl auristatin E payload.	Protein-expression selected ADC strategy; not proof of MET amplification-mediated resistance.
Carboplatin; cisplatin; pemetrexed	Platinum compounds produce DNA crosslinks; pemetrexed inhibits folate-dependent nucleotide synthesis.	Chemotherapy backbones used in EGFR-directed and post-progression combinations.
Paclitaxel; docetaxel; etoposide	Taxanes stabilize microtubules; etoposide inhibits topoisomerase II.	Cytotoxic comparators or lineage-directed regimens; not pathway-selective inhibitors.
Ani9; T16Ainh-A01; CaCCinh-A01	Experimental inhibitors reported to suppress ANO1/TMEM16A-associated calcium-activated chloride conductance, with unequal selectivity.	Tool compounds only; conductance inhibition does not prove tumor dependency or scaffold inhibition.
Niclosamide	Approved anthelmintic with multiple reported cellular targets, including experimental effects on ANO1-related readouts.	Pleiotropy and exposure limitations preclude treating it as selective evidence for ANO1 dependency.
IAA-94; DCPIB	Experimental chloride-associated protein/anion transport blocker (IAA-94) and volume-regulated anion channel tool inhibitor (DCPIB).	Limited selectivity and lack of clinical evidence for EGFR-mutant NSCLC resistance.
TRAM-34; senicapoc	KCa3.1/KCNN4 channel blockers; senicapoc has prior human non-oncology exposure.	Repurposing hypothesis only; no clinical validation for osimertinib-resistant disease.
Astemizole	Histamine H1-receptor antagonist also known to inhibit hERG/KCNH2 and prolong QT.	Cardiac antitarget liability; antitumor experimental effects do not establish a usable oncology window.
Tetrodotoxin; amiloride	Tetrodotoxin blocks susceptible voltage-gated sodium channels; amiloride modulates epithelial Na^+^ transport and at higher concentrations can affect Na^+^/H^+^ exchange.	Nonselective functional probes; no established treatment role in this EGFR-resistance setting.
